# Selection Consistency of Lasso-Based Procedures for Misspecified High-Dimensional Binary Model and Random Regressors

**DOI:** 10.3390/e22020153

**Published:** 2020-01-28

**Authors:** Mariusz Kubkowski, Jan Mielniczuk

**Affiliations:** 1 Institute of Computer Science, Polish Academy of Sciences, Jana Kazimierza 5, 01-248 Warsaw, Poland; m.kubkowski@ipipan.waw.pl; 2 Faculty of Mathematics and Information Science, Warsaw University of Technology, Koszykowa 75, 00-662 Warsaw, Poland

**Keywords:** high-dimensional regression, loss function, random predictors, misspecification, consistent selection, subgaussianity, generalized information criterion, robustness

## Abstract

We consider selection of random predictors for a high-dimensional regression problem with a binary response for a general loss function. An important special case is when the binary model is semi-parametric and the response function is misspecified under a parametric model fit. When the true response coincides with a postulated parametric response for a certain value of parameter, we obtain a common framework for parametric inference. Both cases of correct specification and misspecification are covered in this contribution. Variable selection for such a scenario aims at recovering the support of the minimizer of the associated risk with large probability. We propose a two-step selection Screening-Selection (SS) procedure which consists of screening and ordering predictors by Lasso method and then selecting the subset of predictors which minimizes the Generalized Information Criterion for the corresponding nested family of models. We prove consistency of the proposed selection method under conditions that allow for a much larger number of predictors than the number of observations. For the semi-parametric case when distribution of random predictors satisfies linear regressions condition, the true and the estimated parameters are collinear and their common support can be consistently identified. This partly explains robustness of selection procedures to the response function misspecification.

## 1. Introduction

Consider a random variable $\left(X,Y\right)\in R^{p}\times \left\{0,1\right\}$ and a corresponding response function defined as a posteriori probability q(x)=P(Y=1|X=x). Estimation of the a posteriori probability is of paramount importance in machine learning and statistics since many frequently applied methods, e.g., logistic or tree-based classifiers, rely on it. One of the main estimation methods of *q* is a parametric approach for which the response function is assumed to have parametric form

(1) $$q\left(x\right)=q_{0}\left(\beta^{T}x\right)$$

for some fixed β and known $q_{0}\left(x\right)$. If Equation (1) holds, that is the underlying structure is correctly specified, then it is known that

(2) $$\beta ={\mathrm{argmin}}_{b\in R^{p}}-\{E_{X,Y}\left(Y\log q_{0}\left(b^{T}X\right)+\left(1-Y\right)\log\left(1-q_{0}\left(b^{T}X\right)\right)\right\},$$

or, equivalently (cf., e.g., [1])

(3) $$\beta ={\mathrm{argmin}}_{b}E_{X}KL\left(q\left(X\right),q_{0}\left(X^{T}b\right)\right),$$

where $E_{X}f\left(X\right)$ is the expected value of a random variable f(X) and $KL(q\left(X\right),q_{0}\left(X^{T}b\right))$ is Kullback–Leibler distance between the binary distributions with success probabilities q(X) and $q_{0}\left(X^{T}b\right)$:

$$KL\left(q\left(X\right),q_{0}\left(X^{T}b\right)\right)=q\left(X\right)\log\frac{q(X)}{q_{0}\left(X^{T}b\right)}+\left(1-q\left(X\right)\right)\log\frac{1-q(X)}{1-q_{0}\left(X^{T}b\right)}.$$

The equalities in Equations (2) and (3) form the theoretical underpinning of (conditional) maximum likelihood (ML) method as the expression under the expected value in Equation (2) is the conditional log-likelihood of *Y* given *X* in the parametric model. Moreover, it is a crucial property needed to show that ML estimates of β under appropriate conditions approximate β.

However, more frequently than not, the model in Equation (1) does not hold, i.e., response *q* is misspecified and ML estimators do not approximate β, but the quantity defined by the right-hand side of Equation (3), namely

(4) $$\beta^{*}={\mathrm{argmin}}_{b}E_{X}KL\left(q\left(X\right),q_{0}\left(X^{T}b\right)\right),$$

Thus, parametric fit using conditional ML method, which is the most popular approach to modeling binary response, also has very intuitive geometric and information-theoretic flavor. Indeed, fitting a parametric model, we try to approximate the $\beta^{*}$ which yields averaged KL projection of unknown *q* on set of parametric models ${\left\{q_{0}\left(b^{T}x\right)\right\}}_{b\in R^{p}}$. A typical situation is a semi-parametric framework the true response function satisfies when

(5) $$q\left(x\right)=\tilde{q}\left(\beta^{T}x\right)$$

for some unknown $\tilde{q}\left(x\right)$ and the model in Equation (1) is fitted where $\tilde{q}\neq q_{0}$. An important problem is then how $\beta^{*}$ in Equation (4) relates to β in Equation (5). In particular, a frequently asked question is what can be said about a support of $\beta ={\left(\beta_{1},\ldots ,\beta_{p}\right)}^{T}$, i.e., the set $\left\{i:\beta_{i}\neq 0\right\}$, which consists of indices of predictors which truly influence *Y*. More specifically, an interplay between supports of β and analogously defined support of $\beta^{*}$ is of importance as the latter is consistently estimated and the support of ML estimator is frequently considered as an approximation of the set of true predictors. Variable selection, or equivalently the support recovery of β in high-dimensional setting, is one of the most intensively studied subjects in contemporary statistics and machine learning. This is related to many applications in bioinformatics, biology, image processing, spatiotemporal analysis, and other research areas (see [2,3,4]). It is usually studied under a correct model specification, i.e., under theassumption that data are generated following a given parametric model (e.g., logistic or, in the case of quantitative *Y*, linear model).

Consider the following example: let $\tilde{q}\left(x\right)=q_{L}\left(x^{3}\right)$, where $q_{L}\left(x\right)=e^{x}/\left(1+e^{x}\right)$ is the logistic function. Define regression model by $P\left(Y=1|X\right)=\tilde{q}\left(\beta^{T}X\right)=q_{L}\left({\left(X_{1}+X_{2}\right)}^{3}\right)$, where $X=(X_{1},\ldots ,X_{p})$ is $N(0,I_{p\times p})$-distributed vector of predictors, p>2 and $\beta =\left(1,1,0,\ldots ,0\right)\in R^{p}$. Then, the considered model will obviously be misspecified when the family of logistic models is fitted. However, it turns out in this case that, as *X* is elliptically contoured, $\beta^{*}=\eta \beta =\eta \left(1,1,0,\ldots ,0\right)$ and η≠0 (see [5]) and thus supports of β and $\beta^{*}$ coincide. Thus, in this case, despite misspecification variable selection, i.e., finding out that $X_{1}$ and $X_{2}$ are the only active predictors, it can be solved using the methods described below.

For recent contributions to the study of Kullback–Leibler projections on logistic model (which coincide with Equation (4) for a logistic loss, see below) and references, we refer to the works of Kubkowski and Mielniczuk [6], Kubkowski and Mielniczuk [7] and Kubkowski [8]. We also refer to the work of Lu et al. [9], where the asymptotic distribution of adaptive Lasso is studied under misspecification in the case of fixed number of deterministic predictors. Questions of robustness analysis evolve around an interplay between β and $\beta^{*}$, in particular under what conditions the directions of β and $\beta^{*}$ coincide (cf. the important contribution by Brillinger [10] and Ruud [11]).

In the present paper, we discuss this problem in a more general non-parametric setting. Namely, the minus conditional log-likelihood $-(y\log q_{0}(b^{T}x+\left(1-y\right)\log\left(1-q_{0}\left(b^{T}x\right)\right)$ is replaced by a general loss function of the form

(6) $$l\left(b,x,y\right)=\rho \left(b^{T}x,y\right),$$

where ρ:R×{0,1}→R is some function, $b,x\in R^{p},$ y∈{0,1}, and

$$R\left(b\right)=E_{X,Y}l\left(b,X,Y\right)$$

is the associated risk function for $b\in R^{p}$. Our aim is to determine a support of $\beta^{*}$, where

(7) $$\beta^{*}={\mathrm{argmin}}_{b\in R^{p_{n}}}R\left(b\right).$$

Coordinates of $\beta^{*}$ corresponding to non-zero coefficients are called active predictors and vector $\beta^{*}$ the pseudo-true vector.

The most popular loss functions are related to minus log-likelihood of specific parametric models such as logistic loss

$$l_{logist}\left(b,x,y\right)=-yb^{T}x+\log\left(1+\exp\left(b^{T}x\right)\right)$$

related to $q_{0}\left(b^{T}x\right)=\exp\left(b^{T}x\right)/(1+\exp\left(b^{T}x\right)$, probit loss

$$l_{probit}\left(b,x,y\right)=-y\log\Phi \left(b^{T}x\right)+\left(1-y\right)\log\left(1-\Phi \left(b^{T}x\right)\right)$$

related to $q_{0}\left(b^{T}x\right)=\Phi \left(b^{T}x\right)$, or quadratic loss $l_{lin}\left(b,x,y\right)={\left(y-b^{T}x\right)}^{2}/2$ related to linear regression and quantitative response. Other losses which do not correspond to any parametric model such as Huber loss (see [12]) are constructed with a specific aim to induce certain desired properties of corresponding estimators such as robustness to outliers. We show in the following that variable selection problem can be studied for a general loss function imposing certain analytic properties such as its convexity and Lipschitz property.

For fixed number *p* of predictors smaller than sample size *n*, the statistical consequences of misspecification of a semi-parametric regression model were intensively studied by H. White and his collaborators in the 1980s. The concept of a projection on the fitted parametric model is central to these investigations which show how the distribution of maximum likelihood estimator of $\beta^{*}$ centered by $\beta^{*}$ changes under misspecification (cf. e.g., [13,14]). However, for the case when p>n, the maximum likelihood estimator, which is a natural tool for fixed p≤n case, is ill-defined and a natural question arises: What can be estimated and by what methods?

The aim of the present paper is to study the above problem in high-dimensional setting. To this end, we introduce two-stage approach in which the first stage is based on Lasso estimation (cf., e.g., [2])

(8) $${\hat{\beta }}_{L}={\mathrm{argmin}}_{b\in R^{p_{n}}}\{R_{n}\left(b\right)+\lambda_{L}\sum_{i=1}^{p_{n}}|b_{i}\left|\right\}$$

where $b={\left(b_{1},\ldots ,b_{p_{n}}\right)}^{T}$ and the empirical risk $R_{n}\left(b\right)$ corresponding to R(b) is

$$R_{n}\left(b\right)=n^{-1}\sum_{i=1}^{n}\rho \left(b^{T}X_{i},Y_{i}\right).$$

Parameter $\lambda_{L}>0$ is Lasso penalty, which penalizes large $l_{1}$-norms of potential candidates for a solution. Note that the criterion function in Equation (8) for ρ(s,y)=log(1+exp(−s(2y−1)) can be viewed as penalized empirical risk for the logistic loss. Lasso estimator is thoroughly studied in the case of the linear model when considered loss is square loss (see, e.g., [2,4] for references and overview of the subject) and some of the papers treat the case when such model is fitted to *Y*, which is not necessarily linearly dependent on regressors (cf. [15]). In this case, regression model is misspecified with respect to linear fit. However, similar results are scarce for other scenarios such as logistic fit under misspecification in particular. One of the notable exceptions is Negahban et al. [16], who studied the behavior of Lasso estimate i for a general loss function and possibly misspecified models.

The output of the first stage is Lasso estimate ${\hat{\beta }}_{L}$. The second stage consists in ordering of predictors according to the absolute values of corresponding non-zero coordinates of Lasso estimator and then minimization of Generalized Information Criterion (GIC) on the resulting nested family. This is a variant of SOS (Screening-Ordering-Selection) procedure introduced in [17]. Let ${\hat{s}}^{*}$ be the model chosen by GIC procedure.

Our main contributions are as follows:

- We prove that under misspecification when the sample size grows support ${\hat{s}}^{*}$ coincides with support of $\beta^{*}$ with probability tending to 1. In the general framework allowing for misspecification this means that selection rule ${\hat{s}}^{*}$ is consistent, i.e., $P({\hat{s}}^{*}=s^{*})\to 1$ when n→∞. In particular, when the model in Equation (1) is correctly specified this means that we recover the support of the true vector β with probability tending to 1.
- We also prove approximation result for Lasso estimator when predictors are random and ρ is a convex Lipschitz function (cf. Theorem 1).
- A useful corollary of the last result derived in the paper is determination of sufficient conditions under which active predictors can be separated from spurious ones based on the absolute values of corresponding coordinates of Lasso estimator. This makes construction of nested family containing $s^{*}$ with a large probability possible.
- Significant insight has been gained for fitting of parametric model when predictors are elliptically contoured (e.g., multivariate normal). Namely, it is known that in such situation $\beta^{*}=\eta \beta$, i.e., these two vectors are collinear [5]. Thus, in the case when η≠0 we have that support $s^{*}$ of $\beta^{*}$ coincides with support *s* of β and the selection consistency of two-step procedure proved in the paper entails direction and support recovery of β. This may be considered as a partial justification of a frequent observation that classification methods are robust to misspecification of the model for which they are derived (see, e.g., [5,18]).

We now discuss how our results relate to previous results. Most of the variable selection methods in high-dimensional case are studied for deterministic regressors; here, our results concern random regressors with subgaussian distributions. Note that random regressors scenario is much more realistic for experimental data than deterministic one. The stated results to the best of our knowledge are not available for random predictors even when the model is correctly specified. As to novelty of SS procedure, for its second stage we assume that the number of active predictors is bounded by a deterministic sequence $k_{n}$ tending to infinity and we minimize GIC on family M of models with sizes satisfying also this condition. Such exhaustive search has been proposed in [19] for linear models and extended to GLMs in [20] (cf. [21]). In these papers, GIC has been optimized on all possible subsets of regressors with cardinality not exceeding certain constant $k_{n}$. Such method is feasible for practical purposes only when $p_{n}$ is small. Here, we consider a similar set-up but with important differences: M is a data-dependent small nested family of models and optimization of GIC is considered in the case when the original model is misspecified. The regressors are supposed random and assumptions are carefully tailored to this case. We also stress the fact that the presented results also cover the case when the regression model is correctly specified and Equation (5) is satisfied.

In numerical experiments, we study the performance of grid version of logistic and linear SOS and compare it to its several Lasso-based competitors.

The paper is organized as follows. Section 2 contains auxiliaries, including new useful probability inequalities for empirical risk in the case of subgaussian random variables (Lemma 2). In Section 3, we prove a bound on approximation error for Lasso when the loss function is convex and Lipschitz and regressors are random (Theorem 1). This yields separation property of Lasso. In Theorems 2 and 3 of Section 4, we prove GIC consistency on nested family, which in particular can be built according to the order in which the Lasso coordinates are included in the fitted model. In Section 5.1, we discuss consequences of the proved results for semi-parametric binary model when distribution of predictors satisfies linear regressions condition. In Section 6, we numerically compare the performance of two-stage selection method for two closely related models, one of which is a logistic model and the second one is misspecified.

## 2. Definitions and Auxiliary Results

In the following, we allow random vector (X,Y), q(x), and *p* to depend on sample size *n*, i.e., $\left(X,Y\right)=\left(X^{\left(n\right)},Y^{\left(n\right)}\right)\in R^{p_{n}}\times \left\{0,1\right\}$ and $q_{n}\left(x\right)=P\left(Y^{\left(n\right)}=1|X^{\left(n\right)}=x\right)$. We assume that *n* copies $X_{1}^{\left(n\right)},\ldots ,X_{n}^{\left(n\right)}$ of a random vector $X^{\left(n\right)}$ in $R^{p_{n}}$ are observed together with corresponding binary responses $Y_{1}^{\left(n\right)},\ldots ,Y_{n}^{\left(n\right)}$. Moreover, we assume that observations $(X_{i}^{\left(n\right)},Y_{i}^{\left(n\right)}),\;i=1,\ldots ,n$ are independent and identically distributed (iid). If this condition is satisfied for each *n*, but not necessarily for different *n* and *m*, i.e., distributions of $\left(X_{i}^{\left(n\right)},Y_{i}^{\left(n\right)}\right)$ may be different from that of $\left(X_{j}^{\left(m\right)},Y_{j}^{\left(m\right)}\right)$ or they may be dependent for m≠n, then such framework is called a triangular scenario. A frequently considered scenario is the sequential one. In this case, when sample size *n* increases, we observe values of new predictors additionally to the ones observed earlier. This is a special case of the above scheme as then $X_{i}^{\left(n+1\right)}={\left(X_{i}^{(n)T},X_{i,p_{n}+1},\ldots ,X_{i,p_{n+1}}\right)}^{T}$. In the following, we skip the upper index *n* if no ambiguity arises. Moreover, we write $q\left(x\right)=q_{n}\left(x\right)$. We impose a condition on distributions of random predictors assume that coordinates $X_{ij}$ of $X_{i}$ are subgaussian $Subg(\sigma_{jn}^{2})$ with subgaussianity parameter $\sigma_{jn}^{2}$, i.e., it holds that (see [22])

(9) $$E\exp\left(tX_{ij}\right)\leq \exp\left(t^{2}\sigma_{jn}^{2}/2\right)$$

for all t∈R. This condition basically says that the tails of of $X_{ij}$ do not decrease more slowly than tails of normal distribution $N(0,\sigma_{jn}^{2})$. For future reference, let

$$s_{n}^{2}=\max_{j=1,\ldots ,p_{n}}\sigma_{jn}^{2}$$

and assume in the following that

(10) $$\gamma^{2}:=\underset{n}{lim sup}s_{n}^{2}<\infty .$$

We assume moreover that $X_{i1},\ldots ,X_{ip_{n}}$ are linearly independent in the sense that their arbitrary linear combination is not constant almost everywhere. We consider a general form of response function q(x)=P(Y=1|X=x) and assume that for the given loss function $\beta^{*}$, as defined in Equation (7), exists and is unique. For $s\subseteq \{1,\ldots ,p_{n}\}$, let $\beta^{*}\left(s\right)$ be defined as in Equation (7) when minimum is taken over *b* with support in *s*. We let

$$s^{*}=\mathrm{supp}(\beta^{*}\left(\left\{1,\ldots ,p_{n}\right\}\right)=\left\{i\leq p_{n}:\beta_{i}^{*}\neq 0\right\},$$

denote the support of $\beta^{*}\left(\left\{1,\ldots ,p_{n}\right\}\right)$ with $\beta^{*}\left(\left\{1,\ldots ,p_{n}\right\}\right)={\left(\beta_{1}^{*},\ldots ,\beta_{p_{n}}^{*}\right)}^{T}$.

Let $v_{\pi }={\left(v_{j_{1}},\ldots ,v_{j_{k}}\right)}^{T}\in R^{\left|\pi \right|}$ for $v\in R^{p_{n}}$ and $\pi =\left\{j_{1},\ldots ,j_{k}\right\}\subseteq \left\{1,\ldots ,p_{n}\right\}$. Let $\beta_{s^{*}}^{*}\in R^{|s^{*}|}$ be $\beta^{*}=\beta^{*}\left(\left\{1,\ldots ,p_{n}\right\}\right)$ restricted to its support $s^{*}$. Note that if $s^{*}\subseteq s$, then provided projections are unique (see Section 2) we have

$$\beta_{s^{*}}^{*}=\beta^{*}\left(s^{*}\right)=\beta^{*}{\left(s\right)}_{s^{*}}.$$

Note that this implies that for every superset $s⊇s^{*}$ of *s* the projection $\beta^{*}\left(s\right)$ on the model pertaining to *s* is obtained by appending projection $\beta^{*}\left(s^{*}\right)$ with appropriate number of zeros. Moreover, let

$$\beta_{min}^{*}=\min_{i\in s^{*}}\left|\beta_{i}^{*}\right|.$$

We remark that $\beta^{*}$, $s^{*}$ and $\beta_{min}^{*}$ may depend on *n*. We stress that $\beta_{min}^{*}$ is an important quantity in the development here as it turns out that it may not decrease too quickly in order to obtain approximation results for ${\hat{\beta }}_{L}^{*}$ (see Theorem 1). Note that, when the parametric model is correctly specified, i.e., $q\left(x\right)=q_{0}\left(\beta^{T}x\right)$ for some β with *l* being an associated log-likelihood loss, if *s* is the support of β, we have $s=s^{*}$.

First, we discuss quantities and assumptions needed for the first step of SS procedure.

We consider cones of the form:

(11) $$C_{\varepsilon }=\{\Delta \in R^{p_{n}}:\;||\Delta_{s^{*c}}{\left|\right|}_{1}\leq \left(3+\varepsilon \right)||\Delta_{s^{*}}{\left|\right|}_{1}\},$$

where ε>0, $s^{*c}=\left\{1,\ldots ,p_{n}\right\}\setminus s^{*}$ and $\Delta_{s^{*}}=\left(\Delta_{s_{1}^{*}},\ldots ,\Delta_{s_{|s^{*}|}^{*}}\right)$ for $s^{*}=\left\{s_{1}^{*},\ldots ,s_{|s^{*}|}^{*}\right\}$. Cones $C_{\varepsilon }$ are of special importance because we prove that ${\hat{\beta }}_{L}-\beta^{*}\in C_{\varepsilon }$ (see Lemma 3). In addition, we note that since $l^{1}$-norm is decomposable in the sense that $\left|\right|v_{A}{\left|\right|}_{1}+\left|\right|v_{A^{c}}{\left|\right|}_{1}={\left||v|\right|}_{1}$ the definition of the cone above can be stated as

$$C_{\varepsilon }=\{\Delta \in R^{p_{n}}{:\;||\Delta ||}_{1}\leq \left(4+\varepsilon \right)||\Delta_{s^{*}}{\left|\right|}_{1}\}.$$

Thus, $C_{\varepsilon }$ consists of vectors which do not put too much mass on the complement of $s^{*}$. Let $H\in R^{p_{n}\times p_{n}}$ be a fixed non-negative definite matrix. For cone $C_{\varepsilon }$, we define a quantity $\kappa_{H}\left(\varepsilon \right)$ which can be regarded as a restricted minimal eigenvalue of a matrix in high-dimensional set-up:

(12) $$\kappa_{H}\left(\varepsilon \right)=\underset{\Delta \in C_{\varepsilon }\setminus \left\{0\right\}}{\inf}\frac{\Delta^{T}H\Delta }{\Delta^{T}\Delta }.$$

In the considered context, *H* is usually taken as hessian $D^{2}R\left(\beta^{*}\right)$ and, e.g., for quadratic loss, it equals $EX^{T}X$. When *H* is non-negative definite and not strictly positive definite its smallest eigenvalue $\lambda_{1}=0$ and thus $\inf_{\Delta \in R^{p}\setminus \left\{0\right\}}\frac{\Delta^{T}H\Delta }{\Delta^{T}\Delta }=\lambda_{1}=0$. That is why we have to restrict minimization in Equation (12) in order to have $\kappa_{H}\left(\varepsilon \right)>0$ in the high-dimensional case. As we prove that $\Delta_{0}={\hat{\beta }}_{L}-\beta^{*}\in C_{\varepsilon }$ and would use $0<\kappa_{H}\left(\varepsilon \right)\leq \Delta_{0}^{T}H\Delta_{0}/\Delta_{0}^{T}\Delta_{0}$ it is useful to restrict minimization in Equation (12) to $C_{\varepsilon }\setminus \left\{0\right\}$. Let *R* and $R_{n}$ be the risk and the empirical risk defined above. Moreover, we introduce the following notation:

(13) $$\begin{array}{c} W\left(b\right)=R\left(b\right)-R\left(\beta^{*}\right), \end{array}$$

(14) $$\begin{array}{c} W_{n}\left(b\right)=R_{n}\left(b\right)-R_{n}\left(\beta^{*}\right), \end{array}$$

(15) $$\begin{array}{c} B_{p}\left(r\right)=\{\Delta \in R^{p_{n}}{:\;||\Delta ||}_{p}\leq r\},\;\mathrm{for}\;p=1,2, \end{array}$$

(16) $$\begin{array}{c} S\left(r\right)=\underset{b\in R^{p_{n}}:b-\beta^{*}\in B_{1}\left(r\right)}{\sup}\left|W\left(b\right)-W_{n}\left(b\right)\right|. \end{array}$$

Note that $ER_{n}\left(b\right)=R\left(b\right)$. Thus, S(r) corresponds to oscillation of centred empirical risk over ball $B_{1}\left(r\right)$. We need the following Margin Condition (MC) in Lemma 3 and Theorem 1:

(MC) There exist ϑ,ε,δ>0 and non-negative definite matrix $H\in R^{p_{n}\times p_{n}}$ such that for all *b* with $b-\beta^{*}\in C_{\varepsilon }\cap B_{1}\left(\delta \right)$ we have
  $$R\left(b\right)-R\left(\beta^{*}\right)\geq \frac{\vartheta }{2}{\left(b-\beta^{*}\right)}^{T}H\left(b-\beta^{*}\right).$$

The above condition can be viewed as a weaker version of strong convexity of function *R* (when the right-hand side is replaced by $\vartheta ||b-\beta^{*}{\left|\right|}^{2}$) in the restricted neighbourhood of $\beta^{*}$ (namely, in the intersection of ball $B_{1}\left(\delta \right)$ and cone $C_{\varepsilon }$). We stress the fact that *H* is not required to be positive definite, as in Section 3 we use Condition (MC) together with stronger conditions than $\kappa_{H}\left(\varepsilon \right)>0$ which imply that right hand side of inequality in (MC) is positive. We also do not require here twice differentiability of *R*. We note in particular that Condition (MC) is satisfied in the case of logistic loss, *X* being bounded random variable and $H=D^{2}R\left(\beta^{*}\right)$ (see [23,24,25]). It is also easily seen that that (MC) is satisfied for quadratic loss, *X* such that ${E||X||}_{2}^{2}<\infty$ and $H=D^{2}R\left(\beta^{*}\right)$. Similar condition to (MC) (called Restricted Strict Convexity) was considered in [16] for empirical risk $R_{n}$:

$$R_{n}\left(\beta^{*}+\Delta \right)-R_{n}\left(\beta^{*}\right)\geq DR_{n}{\left(\beta^{*}\right)}^{T}\Delta +\kappa_{L}{\left||\Delta |\right|}^{2}-\tau^{2}\left(\beta^{*}\right)$$

for all $\Delta \in C(3,s^{*})$, some $\kappa_{L}>0$, and tolerance function τ. Note however that MC is a deterministic condition, whereas Restricted Strict Convexity has to be satisfied for random empirical risk function.

Another important assumption, used in Theorem 1 and Lemma 2, is the Lipschitz property of ρ:

(LL) $\exists L>0\;\forall b_{1},b_{2}\in R,y\in \left\{0,1\right\}:\;|\rho \left(b_{1},y\right)-\rho \left(b_{2},y\right)\left|\leq L\right|b_{1}-b_{2}|$.

Now, we discuss preliminaries needed for the development of the second step of SS procedure. Let |w| stand for dimension of *w*. For the second step of the procedure we consider an arbitrary family $M\subseteq 2^{\left\{1,\ldots ,p_{n}\right\}}$ of models (which are identified with subsets of $\left\{1,\ldots ,p_{n}\right\}$ and may be data-dependent) such that $s^{*}\in M,\forall w\in M:\;\left|w\right|\leq k_{n}$ a.e. and $k_{n}\in N_{+}$ is some deterministic sequence. We define Generalized Information Criterion (GIC) as:

(17) $$GIC\left(w\right)=nR_{n}\left(\hat{\beta }\left(w\right)\right)+a_{n}\left|w\right|,$$

where

$$\hat{\beta }\left(w\right)=\underset{b\in R^{p_{n}}:\;b_{w^{c}}=0_{|w^{c}|}}{\mathrm{error}}R_{n}\left(b\right)$$

is ML estimator for model *w* as minimization above is taken over all vectors *b* with support in *w*. Parameter $a_{n}>0$ is some penalty factor depending on the sample size *n* which weighs how important is the complexity of the model described by the number of its variables |w|. Typical examples of $a_{n}$ include:

- AIC (Akaike Information Criterion): $a_{n}=2$;
- BIC (Bayesian Information Criterion): $a_{n}=\log n$; and
- EBIC(*d*) (Extended BIC): $a_{n}=\log n+2d\log p_{n}$, where d>0.

AIC, BIC and EBIC were introduced by Akaike [26], Schwarz [27], and Chen and Chen [19], respectively. Note that for n≥8 BIC penalty is larger than AIC penalty and in its turn EBIC penalty is larger than BIC penalty.

We study properties of $S_{k}\left(r\right)$ for k=1,2, where:

(18) $$S_{k}\left(r\right)=\underset{b\in D_{k}:b-\beta^{*}\in B_{2}\left(r\right)}{\sup}|(W_{n}\left(b\right)-W\left(b\right)|$$

and is the maximal absolute value of the centred empirical risk $W_{n}\left(\cdot \right)$ and sets $D_{k}$ for k=1,2 are defined as follows:

(19) $$\begin{array}{c} D_{1}=\{b\in R^{p_{n}}:\;\exists w\in M:\;|w|\leq k_{n}\wedge s^{*}\subset w\wedge \mathrm{supp}b\subseteq w\}, \end{array}$$

(20) $$\begin{array}{c} D_{2}=\left\{b\in R^{p_{n}}:\;\mathrm{supp}b\subset s^{*}\right\}. \end{array}$$

The idea here is simply to consider sets $D_{i}$ consisting of vectors having no more that $k_{n}$ non-zero coordinates. However, for $s^{*}\leq k_{n}$, we need that for $b\in D_{i}$, we have $|\mathrm{supp}\left(b-\beta^{*}\right)|\leq k_{n}$, what we exploit in Lemma 2. This entails additional condition in the definition of $D_{1}$. Moreover, in Section 4, we consider the following condition $C_{\epsilon }\left(w\right)$ for ϵ>0, $w\subseteq \{1,\ldots ,p_{n}\}$ and some θ>0:

$C_{\epsilon }\left(w\right)$: $R\left(b\right)-R\left(\beta^{*}\right)\geq \theta ||b-\beta^{*}{\left|\right|}_{2}^{2}$ for all $b\in R^{p_{n}}$ such that suppb⊆w and $b-\beta^{*}\in B_{2}\left(\epsilon \right).$

We observe also that, although Conditions (MC) and $C_{\epsilon }\left(w\right)$ are similar, they are not equivalent, as they hold for $v=b-\beta^{*}$ belonging to different sets: $B_{1}\left(r\right)\cap C_{\varepsilon }$ and $B_{2}\left(\epsilon \right)\cap \left\{\Delta \in R^{p_{n}}:\;\mathrm{supp}\Delta \subseteq w\right\}$, respectively. If the minimal eigenvalue $\lambda_{min}$ of matrix *H* in Condition (MC) is positive and Condition (MC) holds for $b-\beta^{*}\in B_{1}\left(r\right)$ (instead of for $b-\beta^{*}\in C_{\varepsilon }\cap B_{1}\left(r\right)$), then we have for $b-\beta^{*}\in B_{2}\left(r/\sqrt{p_{n}}\right)\subseteq B_{1}\left(r\right)$:

$$R\left(b\right)-R\left(\beta^{*}\right)\geq \frac{\vartheta }{2}{\left(b-\beta^{*}\right)}^{T}H\left(b-\beta^{*}\right)\geq \frac{\vartheta \lambda_{min}}{2}||b-\beta^{*}{\left|\right|}_{2}^{2}.$$

Furthermore, if $\lambda_{max}$ is the maximal eigenvalue of *H* and Condition $C_{\epsilon }\left(w\right)$ holds for all $v=b-\beta^{*}\in B_{2}\left(r\right)$ without restriction on suppb, then we have for $b-\beta^{*}\in B_{1}\left(r\right)\subseteq B_{2}\left(r\right)$:

$$R\left(b\right)-R\left(\beta^{*}\right)\geq \theta ||b-\beta^{*}{\left|\right|}_{2}^{2}\geq \frac{\theta }{\lambda_{max}}{\left(b-\beta^{*}\right)}^{T}H\left(b-\beta^{*}\right).$$

Thus, Condition (MC) holds in this case. A similar condition to Condition $C_{\epsilon }\left(w\right)$ for empirical risk $R_{n}$ was considered by Kim and Jeon [28] (formula (2.1)) in the context of GIC minimization. It turns out that Condition $C_{\epsilon }\left(w\right)$ together with ρ(·,y) being convex for all *y* and satisfying Lipschitz Condition (LL) are sufficient to establish bounds which ensure GIC consistency for $k_{n}\ln p_{n}=o\left(n\right)$ and $k_{n}\ln p_{n}=o\left(a_{n}\right)$ (see Corollaries 2 and 3). First, we state the following basic inequality. W(v) and S(r) are defined above the definition of Margin Condition.

**Lemma** **1.** *(Basic inequality). Let ρ(·,y) be convex function for all y. If for some r>0 we have*

$$u=\frac{r}{r+\left|\right|{\hat{\beta }}_{L}-\beta {\left|\right|}_{1}},\;v=u{\hat{\beta }}_{L}+\left(1-u\right)\beta^{*},$$

*then*

$$W\left(v\right)+\lambda ||v-\beta^{*}{\left|\right|}_{1}\leq S\left(r\right)+2\lambda ||v_{s^{*}}-\beta_{s^{*}}^{*}{\left|\right|}_{1}.$$

The proof of the lemma is moved to the Appendix A. It follows from the lemma that, as in view of decomposability of $l^{1}$-distance we have $||v-\beta^{*}{\left|\right|}_{1}=\left|\right|{\left(v-\beta^{*}\right)}_{s}^{*}{\left|\right|}_{1}+\left|\right|{\left(v-\beta^{*}\right)}_{s^{*c}}{\left|\right|}_{1}$, when S(r) is small we have $\left|\right|{\left(v-\beta^{*}\right)}_{s^{*c}}{\left|\right|}_{1}$ is not large in comparison with $\left|\right|{\left(v-\beta^{*}\right)}_{s}^{*}{\left|\right|}_{1}$.

Quantities $S_{k}\left(r\right)$ are defined in Equation (18). Recall that $S_{2}\left(r\right)$ is an oscillation taken over ball $B_{2}\left(r\right)$, whereas $S_{i},i=1,2$ are oscillations taken over $B_{1}\left(r\right)$ ball with restriction on the number of nonzero coordinates.

**Lemma** **2.** *Let ρ(·,y) be convex function for all y and satisfy Lipschitz Condition (LL). Assume that $X_{ij}$ for j≥1 are subgaussian $Subg(\sigma_{jn}^{2})$, where $\sigma_{jn}\leq s_{n}$. Then, for r,t>0:*

*1.*  *$P\left(S\left(r\right)>t\right)\leq \frac{8Lrs_{n}\sqrt{\log(p_{n}\vee 2)}}{t\sqrt{n}}$,*
*2.*  *$P\left(S_{1}\left(r\right)\geq t\right)\leq \frac{8Lrs_{n}\sqrt{k_{n}\ln\left(p_{n}\vee 2\right)}}{t\sqrt{n}}$,*
*3.*  $P\left(S_{2}\left(r\right)\geq t\right)\leq \frac{4Lrs_{n}\sqrt{|s^{*}|}}{t\sqrt{n}}$.

The proof of the Lemma above, which relies on Chebyshev inequality, symmetrization inequality (see Lemma 2.3.1 of [29]), and Talagrand–Ledoux inequality ([30], Theorem 4.12), is moved to the Appendix A. In the case when $\beta^{*}$ does not depend on *n* and thus its support does not change, Part 3 implies in particular that $S_{2}\left(r\right)$ is of the order $n^{-1/2}$ in probability.

## 3. Properties of Lasso for a General Loss Function and Random Predictors

The main result in this section is Theorem 1. The idea for the proof is based on fact that, if S(r) defined in Equation (16) is sufficiently small (condition $S\left(r\right)\leq \bar{C}\lambda r$ is satisfied), then ${\hat{\beta }}_{L}$ lies in a ball $\left\{\Delta \in R^{p_{n}}:\;||\Delta -\beta^{*}|{|}_{1}\leq r\right\}$ (see Lemma 3). Using a tail inequality for S(r) proved in Lemma 2, we obtain Theorem 1. Note that $\kappa_{H}\left(\varepsilon \right)$ has to be bounded away from 0 (condition $2|s^{*}|\lambda \leq \kappa_{H}\left(\varepsilon \right)\vartheta \tilde{C}r$). Convexity of ρ(·,y) below is understood as convexity for both y=0,1.

**Lemma** **3.** Let ρ(·,y) be convex function and assume that λ>0. Moreover, assume margin Condition (MC) with constants ϑ,ϵ,δ>0 and some non-negative definite matrix $H\in R^{p_{n}\times p_{n}}$. If for some r∈(0,δ] we have $S\left(r\right)\leq \bar{C}\lambda r$ and $2|s^{*}|\lambda \leq \kappa_{H}\left(\varepsilon \right)\vartheta \tilde{C}r$, where $\bar{C}=\varepsilon /\left(8+2\varepsilon \right)$ and $\tilde{C}=2/\left(4+\varepsilon \right),$ then

$$\left|\right|{\hat{\beta }}_{L}-\beta^{*}{\left|\right|}_{1}\leq r.$$

The proof of the lemma is moved to the Appendix A.

The first main result provides an exponential inequality for $P\left(\left|\right|{\hat{\beta }}_{L}-\beta^{*}{\left|\right|}_{1}\leq \beta_{min}^{*}/2\right)$. The threshold $\beta_{min}^{*}/2$ is crucial there as it ensures separation: $\max_{i\in s^{*c}}|{\hat{\beta }}_{L,i}|\leq \min_{i\in s^{*}}\left|{\hat{\beta }}_{L,i}\right|$ (see proof of Corollary 1).

**Theorem** **1.** Let ρ(·,y) be convex function for all y and satisfy Lipschitz Condition (LL). Assume that $X_{ij}∼Subg\left(\sigma_{jn}^{2}\right)$, $\beta^{*}$ exists and is unique, margin Condition (MC) is satisfied for ε,δ,ϑ>0, non-negative definite matrix $H\in R^{p_{n}\times p_{n}}$ and let

$$\frac{2|s^{*}|\lambda }{\vartheta \kappa_{H}\left(\varepsilon \right)}\leq \tilde{C}\min\left\{\frac{\beta_{min}^{*}}{2},\delta \right\},$$

*where $\tilde{C}=2/\left(4+\varepsilon \right).$ Then,*

$$P\left(\left|\right|{\hat{\beta }}_{L}-\beta^{*}{\left|\right|}_{1}\leq \frac{\beta_{min}^{*}}{2}\right)\geq 1-2p_{n}e^{-\frac{n\varepsilon^{2}\lambda^{2}}{A}},$$

*where $A=128L^{2}{\left(4+\varepsilon \right)}^{2}s_{n}^{2}$.*

**Proof.**  Let

$$m=\min\left\{\frac{\beta_{min}^{*}}{2},\delta \right\}.$$

Lemmas 2 and 3 imply that:

$$\begin{array}{cc} P\left(\left|\right|{\hat{\beta }}_{L}-\beta^{*}{\left|\right|}_{1}>\frac{\beta_{min}^{*}}{2}\right) & \leq P\left(\left|\right|{\hat{\beta }}_{L}-\beta^{*}{\left|\right|}_{1}>m\right)\leq P\left(S\left(m\right)>\bar{C}\lambda m\right)\\ \; & \leq 2p_{n}e^{-\frac{n\varepsilon^{2}\lambda^{2}}{128L^{2}{\left(4+\varepsilon \right)}^{2}s_{n}^{2}}}. \end{array}$$

 □

**Corollary** **1.** *(Separation property) If assumptions of Theorem 1 are satisfied,*

$$\lambda =\frac{8Ls_{n}\left(4+\varepsilon \right)\phi }{\varepsilon }\sqrt{\frac{2\log(2p_{n})}{n}}$$

for some ϕ>1 and $\kappa_{H}\left(\varepsilon \right)>d$ for some d,ε>0 for large n, $|s^{*}|\lambda =o\left(\min\left\{\beta_{min}^{*},1\right\}\right),$ then

$$P\left(\left|\right|{\hat{\beta }}_{L}-\beta^{*}{\left|\right|}_{1}\leq \frac{\beta_{min}^{*}}{2}\right)\to 1.$$

*Moreover,*

$$P\left(\max_{i\in s^{*c}}|{\hat{\beta }}_{L,i}|\;\leq \;\min_{i\in s^{*}}\left|{\hat{\beta }}_{L,i}\right|\right)\to 1.$$

**Proof.**  The first part of the corollary follows directly from Theorem 1 and the observation that:

$$P\left(\left|\right|{\hat{\beta }}_{L}-\beta^{*}{\left|\right|}_{1}>\frac{\beta_{min}^{*}}{2}\right)\leq e^{\log\left(2p_{n}\right)-\frac{n\varepsilon^{2}\lambda^{2}}{128L^{2}{\left(4+\varepsilon \right)}^{2}s_{n}^{2}}}=e^{\log\left(2p_{n}\right)\left(1-\phi^{2}\right)}\to 0.$$

Now, we prove that condition $\left|\right|{\hat{\beta }}_{L}-\beta^{*}{\left|\right|}_{1}\leq \beta_{min}^{*}/2$ implies separation property

(21) $$\max_{i\in s^{*c}}|{\hat{\beta }}_{L,i}|\leq \min_{i\in s^{*}}\left|{\hat{\beta }}_{L,i}\right|.$$

Indeed, observe that for all $j\in \{1,\ldots ,p_{n}\}$ we have:

(22) $$\frac{\beta_{min}^{*}}{2}\geq \left|\right|{\hat{\beta }}_{L}-\beta^{*}{\left|\right|}_{1}\geq \left|{\hat{\beta }}_{L,j}-\beta_{j}^{*}\right|.$$

If $j\in s^{*},$ then using triangle inequality yields:

$$|{\hat{\beta }}_{L,j}-\beta_{j}^{*}\left|\geq \right|\beta_{j}^{*}\left|-\right|{\hat{\beta }}_{L,j}|\geq \beta_{min}^{*}-\left|{\hat{\beta }}_{L,j}\right|.$$

Hence, from the above inequality and Equation (22), we obtain for $j\in s^{*}$: $|{\hat{\beta }}_{L,j}|\geq \beta_{min}^{*}/2.$ If $j\in s^{*c},$ then $\beta_{j}^{*}=0$ and Equation (22) takes the form: $|{\hat{\beta }}_{L,j}|\leq \beta_{min}^{*}/2.$ This ends the proof. □

We note that the separation property in Equation (21) means that when λ is chosen in an appropriate manner, recovery of $s^{*}$ is feasible with a large probability if all predictors corresponding to absolute value of Lasso coefficient exceeding a certain threshold are chosen. The threshold unfortunately depends on unknown parameters of the model. However, separation property allows to restrict attention to nested family of models and thus to decrease significantly computational complexity of the problem. This is dealt with in the next section. Note moreover that if γ in Equation (10) is finite than λ defined in the Corollary is of order ${\left(\log p_{n}/n\right)}^{1/2}$, which is the optimal order of Lasso penalty in the case of deterministic regressors (see, e.g., [2]).

## 4. GIC Consistency for a a General Loss Function and Random Predictors

Theorems 2 and 3 state probability inequalities related to behavior of GIC on supersets and on subsets of $s^{*}$, respectively. In a nutshell, we show for supersets and subsets separately that the probability that the minimum of GIC is not attained at $s^{*}$ is exponentially small. Corollaries 2 and 3 present asymptotic conditions for GIC consistency in the aforementioned situations. Corollary 4 gathers conclusions of Theorem 1 and Corollaries 1–3 to show consistency of SS procedure (see [17] for consistency of SOS procedure for a linear model with dieterministic predictors) in case of subgaussian variables. Note that in Theorem below we want to consider minimization of GIC in Equation (23) over all supersets of $s^{*}$ as in our applications M is data dependent. As the number of such possible subsets is at least pn−|s*|kn−|s*|, the proof has to be more involved than using reasoning based on Bonferroni inequality.

**Theorem** **2.** *Assume that ρ(·,y) is convex, Lipschitz function with constant L>0, $X_{ij}∼Subg\left(\sigma_{jn}^{2}\right),$ condition $C_{\epsilon }\left(w\right)$ holds for some ϵ,θ>0 and for every $w\subseteq \{1,\ldots ,p_{n}\}$ such that $\left|w\right|\leq k_{n}$. Then, for any r<ϵ, we have:*

(23) $$P\left(\min_{w\in M:s^{*}\subset w}GIC\left(w\right)\leq GIC\left(s^{*}\right)\right)\leq 2p_{n}e^{-\frac{a_{n}^{2}}{k_{n}B}}+2p_{n}e^{-\frac{nD}{k_{n}}},$$

*where $B=32nL^{2}r^{2}k_{n}s_{n}^{2}$ and $D=\theta^{2}r^{2}/512L^{2}s_{n}^{2}$.*

**Proof.**  If $s^{*}\subset w\in M$ and $\hat{\beta }\left(w\right)-\beta^{*}\in B_{2}\left(r\right)$, then in view of inequalities $R_{n}\left(\hat{\beta }\left(s^{*}\right)\right)\leq R_{n}\left(\beta^{*}\right)$ and $R\left(\beta^{*}\right)\leq R\left(b\right)$ we have:

$$\begin{array}{cc} R_{n}\left(\hat{\beta }\left(s^{*}\right)\right)-R_{n}\left(\hat{\beta }\left(w\right)\right) & \leq \underset{b\in D_{1}:b-\beta^{*}\in B_{2}\left(r\right)}{\sup}\left(R_{n}\left(\beta^{*}\right)-R_{n}\left(b\right)\right)\\ \; & \leq \underset{b\in D_{1}:b-\beta^{*}\in B_{2}\left(r\right)}{\sup}\left(\left(R_{n}\left(\beta^{*}\right)-R\left(\beta^{*}\right)\right)-\left(R_{n}\left(b\right)-R\left(b\right)\right)\right)\\ \; & \leq \underset{b\in D_{1}:b-\beta^{*}\in B_{2}\left(r\right)}{\sup}\left|R_{n}\left(b\right)-R\left(b\right)-\left(R_{n}\left(\beta^{*}\right)-R\left(\beta^{*}\right)\right)\right|\\ \; & =S_{1}\left(r\right). \end{array}$$

Note that $a_{n}\left(|w|-\right|s^{*}\left|\right)\geq a_{n}.$ Hence, if we have for some $w⊃s^{*}$: $GIC\left(w\right)\leq GIC\left(s^{*}\right)$, then we obtain $nR_{n}\left(\hat{\beta }\left(s^{*}\right)\right)-nR_{n}\left(\hat{\beta }\left(w\right)\right))\geq a_{n}\left(|w|-\right|s^{*}\left|\right)$ and from the above inequality we have $S_{1}\left(r\right)\geq a_{n}/n$. Furthermore, if $\hat{\beta }\left(w\right)-\beta^{*}\in B_{2}{\left(r\right)}^{c}$ and r<ϵ, then consider:

$$v=u\hat{\beta }\left(w\right)+\left(1-u\right)\beta^{*},$$

where $u=r/(r+||\hat{\beta }\left(w\right)-\beta^{*}|{|}_{2})$. Then

$$||v-\beta^{*}{\left|\right|}_{2}=u||\hat{\beta }\left(w\right)-\beta^{*}{\left|\right|}_{2}=r\cdot \frac{\left|\right|\hat{\beta }\left(w\right)-\beta^{*}{\left|\right|}_{2}}{r+\left|\right|\hat{\beta }\left(w\right)-\beta^{*}{\left|\right|}_{2}}\geq \frac{r}{2},$$

as function x/(x+r) is increasing with respect to *x* for x>0. Moreover, we have $||v-\beta^{*}{\left|\right|}_{2}\leq r<\epsilon$. Hence, in view of $C_{\epsilon }\left(w\right)$ condition, we get:

$$R\left(v\right)-R\left(\beta^{*}\right)\geq \theta ||v-\beta^{*}{\left|\right|}_{2}^{2}\geq \frac{\theta r^{2}}{4}.$$

From convexity of $R_{n}$, we have:

$$R_{n}\left(v\right)\leq u\left(R_{n}\left(\hat{\beta }\left(w\right)\right)-R_{n}\left(\beta^{*}\right)\right)+R_{n}\left(\beta^{*}\right)\leq R_{n}\left(\beta^{*}\right).$$

Let suppv denote the support of vector *v*. We observe that $\mathrm{supp}v\subseteq \mathrm{supp}\hat{\beta }\left(w\right)\cup \mathrm{supp}\beta^{*}\subseteq w$, hence $v\in D_{1}$. Finally, we have:

$$\begin{array}{c} S_{1}\left(r\right)\geq R_{n}\left(\beta^{*}\right)-R\left(\beta^{*}\right)-\left(R_{n}\left(v\right)-R\left(v\right)\right)\geq R\left(v\right)-R\left(\beta^{*}\right)\geq \frac{\theta r^{2}}{4}. \end{array}$$

Hence, we obtain the following sequence of inequalities:

$$\begin{array}{c} P(\min_{w\in M:s^{*}\subset w}GIC\left(w\right)\leq GIC\left(s^{*}\right))\\ \leq P(S_{1}\left(r\right)\geq \frac{a_{n}}{n},\forall w\in M:\;\hat{\beta }\left(w\right)-\beta^{*}\in B_{2}\left(r\right))\\ +P\left(\exists w\in M:s^{*}\subset w\wedge \hat{\beta }\left(w\right)-\beta^{*}\in B_{2}{\left(r\right)}^{c}\right)\leq P\left(S_{1}\left(r\right)\geq \frac{a_{n}}{n}\right)+P\left(S_{1}\left(r\right)\geq \frac{\theta r^{2}}{4}\right)\\ \leq 2p_{n}e^{-\frac{a_{n}^{2}}{32nL^{2}r^{2}k_{n}s_{n}^{2}}}+2p_{n}e^{-\frac{n\theta^{2}r^{2}}{512L^{2}k_{n}s_{n}^{2}}}. \end{array}$$

 □

**Corollary** **2.** *Assume that the conditions of Theorem 2 hold and for some ϵ,θ>0 and for every $w\subseteq \{1,\ldots ,p_{n}\}$ such that $\left|w\right|\leq k_{n}$, $k_{n}\ln\left(p_{n}\vee 2\right)=o\left(n\right)$ and $\underset{n\to \infty }{lim inf}\frac{D_{n}a_{n}}{k_{n}\log\left(2p_{n}\right)}>1$, where $D_{n}^{-1}=128L^{2}s_{n}^{2}\phi /\theta$ for some ϕ>1. Then, we have*

$$P(\min_{w\in M:s^{*}\subset w}GIC\left(w\right)\leq GIC\left(s^{*}\right))\to 0.$$

**Proof.**  We the choose allb radius *r* of $B_{2}\left(r\right)$ in a special way. Namely, we take:

$$r_{n}^{2}=\frac{512\phi^{2}L^{2}s_{n}^{2}\log\left(2p_{n}\right)k_{n}}{n\theta^{2}}$$

for some ϕ>1. In view of assumptions $r_{n}\to 0$. Consider $n_{0}$ such that $r_{n}<\epsilon$ for all $n\geq n_{0}$. Hence, the second term of the upper bound in Equation (23) for $r=r_{n}$ is equal to:

$$2p_{n}e^{-\frac{n\theta^{2}r_{n}^{2}}{512L^{2}k_{n}s_{n}^{2}}}=e^{\log\left(2p_{n}\right)\left(1-\phi^{2}\right)}\to 0.$$

Similarly, the first term of the upper bound in Equation (23) is equal to:

$$2p_{n}e^{-\frac{a_{n}^{2}}{32nL^{2}r_{n}^{2}k_{n}s_{n}^{2}}}=e^{\log\left(2p_{n}\right)\left(1-\frac{a_{n}^{2}\theta^{2}}{128^{2}L^{4}k_{n}^{2}s_{n}^{4}\phi^{2}\log^{2}\left(2p_{n}\right)}\right)}=e^{\log\left(2p_{n}\right)\left(1-\frac{D_{n}^{2}a_{n}^{2}}{k_{n}^{2}\log^{2}\left(2p_{n}\right)}\right)}\to 0.$$

These two convergences end the proof. □

The most restrictive condition of Corollary 2 is $\underset{n\to \infty }{lim inf}\frac{D_{n}a_{n}}{k_{n}\log\left(2p_{n}\right)}>1$ which is slightly weaker than $k_{n}\ln\left(p_{n}\vee 2\right)=o\left(a_{n}\right)$. The following remark proved in the Appendix A gives sufficient conditions for consistency of BIC and EBIC penalties, which do not satisfy condition $k_{n}\log\left(p_{n}\right)=o\left(a_{n}\right)$.

**Remark** **1.** *If in Corollary 2 we assume $D_{n}\geq A$ for some A>0, then condition $\underset{n\to \infty }{lim inf}\frac{D_{n}a_{n}}{k_{n}\log\left(2p_{n}\right)}>1$ holds when:*

*(1)*  *$a_{n}=\log n$ and $p_{n}<\frac{n^{\frac{A}{k_{n}\left(1+u\right)}}}{2}$ for some u>0.*
*(2)*  *$a_{n}=\log n+2\gamma \log p_{n}$, $k_{n}\leq C$ and 2Aγ−(1+u)C≥0, where C,u>0.*
*(3)*  *$a_{n}=\log n+2\gamma \log p_{n}$, $k_{n}\leq C$, 2Aγ−(1+u)C<0, $p_{n}<Bn^{\delta }$, where $\delta =\frac{A}{(1+u)C-2A\gamma }$ and $B=2^{-(1+u)C}$.*

Theorem 3 is an analog of Theorem 2 for subsets of $s^{*}$.

**Theorem** **3.** Assume that ρ(·,y) is convex, Lipschitz function with constant L>0, $X_{ij}∼Subg\left(\sigma_{jn}^{2}\right),$ condition $C_{\epsilon }\left(s^{*}\right)$ holds for some ϵ,θ>0, and $8a_{n}\left|s^{*}\right|\leq \theta n\min\left\{\epsilon^{2},\beta_{min}^{*2}\right\}$. Then, we have:

$$P\left(\min_{w\in M:w\subset s^{*}}GIC\left(w\right)\leq GIC\left(s^{*}\right)\right)\leq \sqrt{2}e^{-n\min{\left\{\epsilon ,\beta_{min}^{*}\right\}}^{2}E},$$

where $E=\theta^{2}/2^{12}L^{2}s_{n}^{2}\left|s^{*}\right|$

**Proof.**  Suppose that for some $w\subset s^{*}$ we have $GIC\left(w\right)\leq GIC\left(s^{*}\right)$. This is equivalent to:

$$nR_{n}\left(\hat{\beta }\left(s^{*}\right)\right)-nR_{n}\left(\hat{\beta }\left(w\right)\right)\geq a_{n}\left(|w|-\right|s^{*}\left|\right).$$

In view of inequalities $R_{n}\left(\hat{\beta }\left(s^{*}\right)\right)\leq R_{n}\left(\beta^{*}\right)$ and $a_{n}\left(|w|-\right|s^{*}\left|\right)\geq -a_{n}\left|s^{*}\right|$, we obtain:

$$nR_{n}\left(\beta^{*}\right)-nR_{n}\left(\hat{\beta }\left(w\right)\right)\geq -a_{n}\left|s^{*}\right|.$$

Let $v=u\hat{\beta }\left(w\right)+\left(1-u\right)\beta^{*}$ for some u∈[0,1] to be specified later. From convexity of ρ, we consider:

(24) $$nR_{n}\left(\beta^{*}\right)-nR_{n}\left(v\right)\geq nu\left(R_{n}\left(\beta^{*}\right)-R_{n}\left(\hat{\beta }\left(w\right)\right)\right)\geq -ua_{n}|s^{*}|\geq -a_{n}\left|s^{*}\right|.$$

We consider two cases separately:

(1) $\beta_{min}^{*}>\epsilon$.

First, observe that

(25) $$8a_{n}\left|s^{*}\right|\leq \theta \epsilon^{2}n,$$

which follows from our assumption. Let $u=\epsilon /(\epsilon +||\hat{\beta }\left(w\right)-\beta^{*}|{|}_{2})$ and

(26) $$v=u\hat{\beta }\left(w\right)+\left(1-u\right)\beta^{*}.$$

Note that $\left|\right|\hat{\beta }\left(w\right)-\beta^{*}{\left|\right|}_{2}\geq \left|\right|\beta_{s^{*}\setminus w}^{*}{\left|\right|}_{2}\geq \beta_{min}^{*}$. Then, as function d(x)=x/(x+c) is increasing and bounded from above by 1 for x,c>0, we obtain:

(27) $$\epsilon \geq ||v-\beta^{*}{\left|\right|}_{2}=\frac{\epsilon ||\hat{\beta }\left(w\right)-\beta^{*}{\left|\right|}_{2}}{\epsilon +\left|\right|\hat{\beta }\left(w\right)-\beta^{*}{\left|\right|}_{2}}\geq \frac{\epsilon \beta_{min}^{*}}{\epsilon +\beta_{min}^{*}}>\frac{\epsilon^{2}}{2\epsilon }=\frac{\epsilon }{2}.$$

Hence, in view of $C_{\epsilon }\left(s^{*}\right)$ condition, we have:

$$R\left(v\right)-R\left(\beta^{*}\right)>\theta \frac{\epsilon^{2}}{4}.$$

Using Equations (24)–(26) and the above inequality yields:

$$\begin{array}{c} S_{2}\left(\epsilon \right)\geq R_{n}\left(\beta^{*}\right)-R\left(\beta^{*}\right)-\left(R_{n}\left(v\right)-R\left(v\right)\right)>\theta \frac{\epsilon^{2}}{4}-\frac{a_{n}}{n}\left|s^{*}\right|\geq \frac{\theta \epsilon^{2}}{8}. \end{array}$$

Thus, in view of Lemma 2, we obtain:

(28) $$P\left(\min_{w\in M:w\subset s^{*}}GIC\left(w\right)\leq GIC\left(s^{*}\right)\right)\leq P\left(S_{2}\left(\epsilon \right)>\frac{\theta \epsilon^{2}}{8}\right)\leq \sqrt{2}e^{-\frac{n\theta^{2}\epsilon^{2}}{4096L^{2}s_{n}^{2}\left|s^{*}\right|}}.$$

(2) $\beta_{min}^{*}\leq \epsilon$.

In this case, we take $u=\beta_{min}^{*}/(\beta_{min}^{*}+\left|\right|\hat{\beta }\left(w\right)-\beta^{*}{\left|\right|}_{2})$ and define *v* as in Equation (26). Analogously, as in Equation (27), we have:

$$\frac{\beta_{min}^{*}}{2}\leq ||v-\beta^{*}{\left|\right|}_{2}\leq \beta_{min}^{*}.$$

Hence, in view of $C_{\epsilon }\left(s^{*}\right)$ condition, we have:

$$R\left(v\right)-R\left(\beta^{*}\right)\geq \theta \frac{\beta_{min}^{*2}}{4}.$$

Using Equation (24) and the above inequality yields:

$$\begin{array}{c} S_{2}\left(\beta_{min}^{*}\right)\geq R_{n}\left(\beta^{*}\right)-R\left(\beta^{*}\right)-\left(R_{n}\left(v\right)-R\left(v\right)\right)\geq \theta \frac{\beta_{min}^{*2}}{4}-\frac{a_{n}}{n}\left|s^{*}\right|\geq \frac{\theta }{8}\beta_{min}^{*2}. \end{array}$$

Thus, in view of Lemma 2, we obtain:

(29) $$P\left(\min_{w\in M:w\subset s^{*}}GIC\left(w\right)\leq GIC\left(s^{*}\right)\right)\leq P\left(S_{2}\left(\beta_{min}^{*}\right)\geq \frac{\theta }{8}\beta_{min}^{*2}\right)\leq \sqrt{2}e^{-\frac{n\theta^{2}\beta_{min}^{*2}}{2^{12}L^{2}s_{n}^{2}\left|s^{*}\right|}}.$$

By combining Equations (28) and (29), the theorem follows. □

**Corollary** **3.** Assume that loss ρ(·,y) is convex, Lipschitz function with constant L>0, $X_{ij}∼Subg\left(\sigma_{jn}^{2}\right),$ condition $C_{\epsilon }\left(s^{*}\right)$ holds for some ϵ,θ>0 and $a_{n}\left|s^{*}\right|=o\left(n\min{\left\{1,\beta_{min}^{*}\right\}}^{2}\right)$, then

$$P(\min_{w\in M:w\subset s^{*}}GIC\left(w\right)\leq GIC\left(s^{*}\right))\to 0.$$

**Proof.**  First, observe that as $a_{n}\to \infty$

$$a_{n}\left|s^{*}\right|=o\left(n\min{\left\{1,\beta_{min}^{*}\right\}}^{2}\right)$$

implies

$$|s^{*}|=o\left(n\min{\left\{1,\beta_{min}^{*}\right\}}^{2}\right),$$

and thus in view of Theorem 3 we have

$$P(\min_{w\in M:w\subset s^{*}}GIC\left(w\right)\leq GIC\left(s^{*}\right))\to 0.$$

## 5. Selection Consistency of SS Procedure

In this section, we combine the results of the two previous sections to establish consistency of a two-step SS procedure. It consists in construction of a nested family of models M using magnitude of Lasso coefficients and then finding the minimizer of GIC over this family. As M is data dependent to establish consistency of the procedure we use Corollaries 2 and 3 in which the minimizer of GIC is considered over *all* subsets and supersets of $s^{*}$.

SS (Screening and Selection) procedure is defined as follows:

1. Choose some λ>0.
2. Find ${\hat{\beta }}_{L}=\underset{b\in R^{p_{n}}}{arg min}R_{n}\left(b\right)+{\lambda ||b||}_{1}$.
3. Find ${\hat{s}}_{L}=\mathrm{supp}{\hat{\beta }}_{L}=\left\{j_{1},\ldots ,j_{k}\right\}$ such that $|{\hat{\beta }}_{L,j_{1}}\left|\geq \ldots \geq \right|{\hat{\beta }}_{L,j_{k}}|>0$ and $j_{1},\ldots ,j_{k}\in \left\{1,\ldots ,p_{n}\right\}$.
4. Define $M_{SS}=\left\{\emptyset ,\left\{j_{1}\right\},\left\{j_{1},j_{2}\right\},\ldots ,\left\{j_{1},j_{2},\ldots ,j_{k}\right\}\right\}$.
5. Find ${\hat{s}}^{*}=\underset{w\in M_{SS}}{arg min}GIC\left(w\right)$.

The SS procedure is a modification of SOS procedure in [17] designed for linear models. Since ordering step considered in [17] is omitted in the proposed modification, we abbreviate the name to SS.

Corollary 4 and Remark 2 describe the situations when SS procedure is selection consistent. In it, we use the assumptions imposed in Section 2 and Section 3 together with an assumption that support of $s^{*}$ contains no more than $k_{n}$ elements, where $k_{n}$ is some deterministic sequence of integers. Let $M_{SS}$ is nested family constructed in the step 4 of SS procedure.

**Corollary** **4.** Assume that ρ(·,y) is convex, Lipschitz function with constant L>0, $X_{ij}∼Subg\left(\sigma_{jn}^{2}\right)$ and $\beta^{*}$ exists and is unique. If $k_{n}\in N_{+}$ is some sequence, margin Condition (MC) is satisfied for some ϑ,δ,ε>0, condition $C_{\epsilon }\left(w\right)$ holds for some ϵ,θ>0 and for every $w\subseteq \{1,\ldots ,p_{n}\}$ such that $\left|w\right|\leq k_{n}$ and the following conditions are fulfilled:

- $|s^{*}|\leq k_{n}$,
- *$P(\forall w\in M_{SS}:|w|\leq k_{n})\to 1$,*
- *$\underset{n}{lim inf}\kappa_{H}\left(\varepsilon \right)>0$ for some ε>0, where H is non-negative definite matrix and $\kappa_{H}\left(\varepsilon \right)$ is defined in Equation (12),*
- *$\log\left(p_{n}\right)=o\left(n\lambda^{2}\right)$,*
- $k_{n}\lambda =o\left(\min\left\{\beta_{min}^{*},1\right\}\right)$,
- *$k_{n}\log p_{n}=o\left(n\right)$,*
- *$k_{n}\log p_{n}=o\left(a_{n}\right)$,*
- $a_{n}k_{n}=o\left(n\min{\left\{\beta_{min}^{*},1\right\}}^{2}\right)$,

*then for SS procedure we have*

$$P({\hat{s}}^{*}=s^{*})\to 1.$$

**Proof.**  In view of Corollary 1, following from the separation property in Equation (22) we obtain $P(s^{*}\in M_{SS})\to 1$. Let:

$$\begin{array}{c} A_{1}=\left\{\min_{w\in M_{SS}:w⊃s^{*},\left|w\right|\leq k_{n}}GIC\left(w\right)\leq GIC\left(s^{*}\right)\right\},\\ A_{2}=\left\{\min_{w\in M_{SS}:w⊃s^{*},\left|w\right|>k_{n}}GIC\left(w\right)\leq GIC\left(s^{*}\right)\right\},\\ B=\{\forall w\in M_{SS}:|w|\leq k_{n}\}. \end{array}$$

Then, we have again from the fact that $A_{2}\cap B=\emptyset$, union inequality and Corollary 2:

(30) $$\begin{array}{cc} P(\min_{w\in M_{SS}:w⊃s^{*}}GIC\left(w\right)\leq GIC\left(s^{*}\right)) & =P\left(A_{1}\cup A_{2}\right)=P\left(A_{1}\cup \left(A_{2}\cap B^{c}\right)\right)\\ \; & \leq P\left(A_{1}\right)+P\left(B^{c}\right)\to 0. \end{array}$$

In an analogous way, using $|s^{*}|\leq k_{n}$ and Corollary 3 yields:

(31) $$P(\min_{w\in M_{SS}:w\subset s^{*}}GIC\left(w\right)\leq GIC\left(s^{*}\right))\to 0.$$

Now, observe that in view of definition of ${\hat{s}}^{*}$ and union inequality:

$$\begin{array}{c} P\left({\hat{s}}^{*}=s^{*}\right)=P\left(\min_{w\in M_{SS}:w\neq s^{*}}GIC\left(w\right)>GIC\left(s^{*}\right)\right)\\ \geq 1-P(\min_{w\in M_{SS}:w\subset s^{*}}GIC\left(w\right)\leq GIC\left(s^{*}\right))\\ -P(\min_{w\in M_{SS}:w⊃s^{*}}GIC\left(w\right)\leq GIC\left(s^{*}\right)). \end{array}$$

Thus, $P({\hat{s}}^{*}=s^{*})\to 1$ in view of the above inequality and Equations (30) and (31). □

### 5.1. Case of Misspecified Semi-Parametric Model

Consider now the important case of the misspecified semi-parametric model defined in Equation (5) for which function $\tilde{q}$ is unknown and may be arbitrary. An interesting question is whether information about β can be recovered when misspecification occurs. The answer is positive under some additional assumptions on distribution of random predictors. Assume additionally that *X* satisfies

(32) $$E\left(X|\beta^{T}X\right)=u_{0}+u\beta^{T}X,$$

where β is the true parameter. Thus, regressions of *X* given $\beta^{T}X$ have to be linear. We stress that conditioning $\beta^{T}X$ involves only the true β in Equation (5). Then, it is known (cf. [5,10,11]) that $\beta^{*}=\eta \beta$ and η≠0 if Cov(Y,X)≠0. Note that because β and $\beta^{*}$ are collinear and η≠0 it follows that $s=s^{*}$. This is important in practical applications as it shows that a position of the optimal separating direction given by β can be consistently recovered. It is also worth mentioning that if Equation (32) is satisfied the direction of β coincides with the direction of the first canonical vector. We refer to the work of Kubkowski and Mielniczuk [7] for the proof and to the work o Kubkowski and Mielniczuk [6] for discussion and up-to date references to this problem. The linear regressions condition in Equation (32) is satisfied, e.g., by elliptically contoured distribution, in particular by multivariate normal. We note that it is proved in [18] that Equation (32) approximately holds for the majority of β. When Equation (32) holds exactly, proportionality constant η can be calculated numerically for known $\tilde{q}$ and β. We can state thus the following result provided Equation (32) is satisfied.

**Corollary** **5.** Assume that Equation (32) and the assumptions of Corollary 4 are satisfied. Moreover, Cov(Y,X)≠0. Then, $P({\hat{s}}^{*}=s)\to 1$.

**Remark** **2.** If $p_{n}=O\left(e^{cn^{\gamma }}\right)$ for some c>0, γ∈(0,1/2), ξ∈(0,0.5−γ), u∈(0,0.5−γ−ξ), $k_{n}=O\left(n^{\xi }\right)$, $\lambda =C_{n}\sqrt{\log(p_{n})/n}$, $C_{n}=O\left(n^{u}\right)$, $C_{n}\to +\infty$, $n^{-\frac{\gamma }{2}}=O\left(\beta_{min}^{*}\right)$, $a_{n}=dn^{\frac{1}{2}-u}$, then assumptions imposed on asymptotic behavior of parameters in Corollary 4 are satisfied.

Note that $p_{n}$ is allowed to grow exponentially: $\log p_{n}=O\left(n^{\gamma }\right)$, however $\beta_{min}^{*}$ may not decrease to 0 too quickly with regard to growth of $p_{n}$: $n^{-\frac{\gamma }{2}}=O\left(\beta_{min}^{*}\right)$.

**Remark** **3.** We note that, to apply Corollary 4 to the two-step procedure based on Lasso, it is required that $|s^{*}|\leq k_{n}$ and that the support of Lasso estimator with probability tending to 1 contains no more than $k_{n}$ elements. Some results bounding $|\mathrm{supp}{\hat{\beta }}_{L}|$ are available for deterministic X (see [31]) and for random X (see [32]), but they are too weak to be useful for EBIC penalties. The other possibility to prove consistency of two-step procedure is to modify it in the first step by using thresholded Lasso (see [33]) corresponding to $k_{n}^{\prime }$ largest Lasso coefficients where $k_{n}^{\prime }\in N$ is such that $k_{n}=o\left(k_{n}^{\prime }\right)$. This is a subject of ongoing research.

## 6. Numerical Experiments

### 6.1. Selection Procedures

We note that the original procedure is defined for a single λ only. In the simulations discussed below, we implemented modifications of SS procedure introduced in Section 5. In practice, it is generally more convenient to consider in the first step some sequence of penalty parameters $\lambda_{1}>\ldots >\lambda_{m}>0$ instead of only one λ in order to avoid choosing the “best” λ. For the fixed sequence $\lambda_{1},\ldots ,\lambda_{m}$, we construct corresponding families $M_{1},\ldots ,M_{m}$ analogously to M in Step 4 of the SS procedure. Thus, we arrive at the following SSnet procedure, which is the modification of SOSnet procedure in [17]. Below, $\tilde{b}$ is a vector *b* with first coordinate corresponding to intercept omitted, $b={\left(b_{0},{\tilde{b}}^{T}\right)}^{T}$:

1. Choose some $\lambda_{1}>\ldots >\lambda_{m}>0$.
2. Find ${\hat{\beta }}_{L}^{\left(i\right)}=\underset{b\in R^{p_{n}+1}}{arg min}R_{n}\left(b\right)+\lambda_{i}\left|\right|\tilde{b}{\left|\right|}_{1}$ for i=1,…,m.
3. Find ${\hat{s}}_{L}^{\left(i\right)}=\mathrm{supp}{\hat{\tilde{\beta }}}_{L}^{\left(i\right)}=\left\{j_{1}^{\left(i\right)},\ldots ,j_{k_{i}}^{\left(i\right)}\right\}$ where $j_{1}^{\left(i\right)},\ldots ,j_{k_{i}}^{\left(i\right)}$ are such that $|{\hat{\beta }}_{L,j_{1}^{\left(i\right)}}^{\left(i\right)}\left|\geq \ldots \geq \right|{\hat{\beta }}_{L,j_{k_{i}}^{\left(i\right)}}^{\left(i\right)}|>0$ for i=1,…,m.
4. Define $M_{i}=\left\{\left\{j_{1}^{\left(i\right)}\right\},\left\{j_{1}^{\left(i\right)},j_{2}^{\left(i\right)}\right\},\ldots ,\left\{j_{1}^{\left(i\right)},j_{2}^{\left(i\right)},\ldots ,j_{k_{i}}^{\left(i\right)}\right\}\right\}$ for i=1,…,m.
5. Define $M=\left\{\emptyset \right\}\cup \overset{m}{\underset{i=1}{⋃}}M_{i}$.
6. Find ${\hat{s}}^{*}=\underset{w\in M}{arg min}GIC\left(w\right)$, where
  $$GIC\left(w\right)=\min_{b\in R^{p_{n}+1}:\mathrm{supp}\tilde{b}\subseteq w}nR_{n}\left(b\right)+a_{n}\left(|w|+1\right).$$

Instead of constructing families $M_{i}$ for each $\lambda_{i}$ in SSnet procedure, λ can be chosen by cross-validation using 1SE rule (see [34]) and then SS procedure is applied for such λ. We call this procedure SSCV. The last procedure considered was introduced by Fan and Tang [35] and is Lasso procedure with penalty parameter $\hat{\lambda }$ chosen in a data-dependent way analogously to SSCV. Namely, it is the minimizer of GIC criterion with $a_{n}=\log\left(\log n\right)\cdot \log p_{n}$ for which ML estimator has been replaced by Lasso estimator with penalty λ. Once ${\hat{\beta }}_{L}\left({\hat{\lambda }}_{L}\right)$ is calculated, then ${\hat{s}}^{*}$ is defined as its support. The procedure is called LFT in the sequel.

We list below versions of the above procedures along with R packages that were used to choose sequence $\lambda_{1},\ldots ,\lambda_{m}$ and computation of Lasso estimator. The following packages were chosen based on selection performance after initial tests for each loss and procedure:

- SSnet with logistic or quadratic loss: ncvreg;
- SSCV or LFT with logistic or quadratic loss: glmnet; and
- SSnet, SSCV or LFT with Huber loss (cf. [12]): hqreg.

The following functions were used to optimize $R_{n}$ in GIC minimization step for each loss:

- logistic loss: glm.fit (package stats);
- quadratic loss: .lm.fit (package stats); and
- Huber loss: rlm (package rlm).

Before applying the investigated procedures, each column of matrix $X={\left(X_{1},\ldots ,X_{n}\right)}^{T}$ was standardized as Lasso estimator ${\hat{\beta }}_{L}$ depends on scaling of predictors. We set length of $\lambda_{i}$ sequence to m=20. Moreover, in all procedures we considered only $\lambda_{i}$ for which $|{\hat{s}}_{L}^{\left(i\right)}|\leq n$ because, when $|{\hat{s}}_{L}^{\left(i\right)}|>n$, Lasso and ML solutions are not unique (see [32,36]). For Huber loss, we set parameter δ=1/10 (see [12]). The number of folds in SSCV was set to K=10.

Each simulation run consisted of *L* repetitions, during which samples $X_{k}={\left(X_{1}^{\left(k\right)},\ldots ,X_{n}^{\left(k\right)}\right)}^{T}$ and $Y_{k}={\left(Y_{1}^{\left(k\right)},\ldots ,Y_{n}^{\left(k\right)}\right)}^{T}$ were generated for k=1,…,L. For *k*th sample $\left(X_{k},Y_{k}\right)$ estimator ${\hat{s}}_{k}^{*}$ of set of active predictors was obtained by a given procedure as the support of $\hat{\tilde{\beta }}\left({\hat{s}}_{k}^{*}\right)$, where

$$\hat{\beta }\left({\hat{s}}_{k}^{*}\right)={\left({\hat{\beta }}_{0}\left({\hat{s}}_{k}^{*}\right),\hat{\tilde{\beta }}{\left({\hat{s}}_{k}^{*}\right)}^{T}\right)}^{T}=\underset{b\in R^{p_{n}+1}}{arg min}\frac{1}{n}\sum_{i=1}^{n}\rho \left(b^{T}X_{i}^{\left(k\right)},Y_{i}^{\left(k\right)}\right)$$

is ML estimator for *k*th sample. We denote by $M^{\left(k\right)}$ the family M obtained by a given procedure for *k*th sample.

In our numerical experiments we have computed the following measures of selection performance which gauge co-direction of true parameter β and $\hat{\beta }$ and the interplay between $s^{*}$ and ${\hat{s}}^{*}$:

- $ANGLE=\frac{1}{L}\sum_{k=1}^{L}\arccos\left|\cos∠\left({\tilde{\beta }}_{0},\hat{\tilde{\beta }}\left({\hat{s}}_{k}^{*}\right)\right)\right|$, where
  $$\cos∠\left(\tilde{\beta },\hat{\tilde{\beta }}\left({\hat{s}}_{k}^{*}\right)\right)=\frac{\sum_{j=1}^{p_{n}}\beta_{j}{\hat{\beta }}_{j}\left({\hat{s}}_{k}^{*}\right)}{\left|\right|\tilde{\beta }{\left|\right|}_{2}\left|\right|\hat{\tilde{\beta }}\left({\hat{s}}_{k}^{*}\right){\left|\right|}_{2}}$$
  and we let $\cos∠(\tilde{\beta },\hat{\tilde{\beta }}\left({\hat{s}}_{k}^{*}\right))=0$, if $\left|\right|\tilde{\beta }{\left|\right|}_{2}\left|\right|\hat{\tilde{\beta }}\left({\hat{s}}_{k}^{*}\right){\left|\right|}_{2}=0$,
- $P_{inc}=\frac{1}{L}\sum_{k=1}^{L}I\left(s^{*}\in M^{\left(k\right)}\right)$,
- $P_{equal}=\frac{1}{L}\sum_{k=1}^{L}I\left({\hat{s}}_{k}^{*}=s^{*}\right)$.
- $P_{supset}=\frac{1}{L}\sum_{k=1}^{L}I\left({\hat{s}}_{k}^{*}⊇s^{*}\right)$.

Thus, ANGLE is equal an of angle between true parameter (with intercept omitted) and its post model-selection estimator averaged over simulations, $P_{inc}$ is a fraction of simulations for which family $M^{\left(k\right)}$ contains true model $s^{*}$, and $P_{equal}$ and $P_{supset}$ are the fractions of time when SSnet chooses true model or its superset, respectively.

### 6.2. Regression Models Considered

To investigate behavior of two-step procedure under misspecification we considered two similar models with different sets of predictors. As sets of predictors differ, this results in correct specification of the first model (Model M1) and misspecification of the second (Model M2).

Namely, in Model M1, we generated *n* observations $\left(X_{i},Y_{i}\right)\in R^{p+1}\times \left\{0,1\right\}$ for i=1,…,n such that:

$$\begin{array}{c} X_{i0}=1,X_{i1}=Z_{i1},X_{i2}=Z_{i2},X_{ij}=Z_{i,j-7}\;\mathrm{for}\;j=10,\ldots ,p,\\ X_{i3}=X_{i1}^{2},X_{i4}=X_{i2}^{2},X_{i5}=X_{i1}X_{i2},\\ X_{i6}=X_{i1}^{2}X_{i2},X_{i7}=X_{i1}X_{i2}^{2},X_{i8}=X_{i1}^{3},X_{i9}=X_{i2}^{3}, \end{array}$$

where $Z_{i}={\left(Z_{i1},\ldots ,Z_{ip}\right)}^{T}∼N_{p}\left(0_{p},\Sigma \right)$, $\Sigma ={\left[\rho^{\left|i-j\right|}\right]}_{i,j=1,\ldots ,p}$ and ρ∈(−1,1). We consider response function $q\left(x\right)=q_{L}\left(x^{3}\right)$ for x∈R, s={1,2} and $\beta_{s}={\left(1,1\right)}^{T}$. Thus,

$$\begin{array}{cc} P(Y_{i}=1|X_{i}=x_{i}) & =q\left(\beta_{s}^{T}x_{i,s}\right)=q\left(x_{i1}+x_{i2}\right)=q_{L}\left({\left(x_{i1}+x_{i2}\right)}^{3}\right)\\ \; & =q_{L}\left(x_{i1}^{3}+x_{i2}^{3}+3x_{i1}^{2}x_{i2}+3x_{i1}x_{i2}^{2}\right)\\ \; & =q_{L}\left(3x_{i6}+3x_{i7}+x_{i8}+x_{i9}\right). \end{array}$$

We observe that the last equality implies that the above binary model is correctly specified with respect to family of fitted logistic models and $X_{6},X_{7},X_{8}$ and $X_{9}$ are four active predictors, whereas the remaining ones play no role in prediction of *Y*. Hence, $s^{*}=\left\{6,7,8,9\right\}$ and $\beta_{s^{*}}^{*}={\left(3,3,1,1\right)}^{T}$ are, respectively, sets of indices of active predictors and non-zero coefficients of projection onto family of logistic models.

We considered the following parameters in numerical experiments: n=500,p=150,ρ∈{−0.9+0.15·k:k=0,1,…,12}, and L=500 (the number of generated datasets for each combination of parameters). We investigated procedures SSnet, SSCV, and LFT using logistic, quadratic, and Huber (cf. [12]) loss functions. For procedures SSnet and SSCV, we used GIC penalties with:

- $a_{n}=\log n$ (BIC); and
- $a_{n}=\log n+2\log p_{n}$ (EBIC1).

In Model M2, we generated *n* observations $\left(X_{i},Y_{i}\right)\in R^{p+1}\times \left\{0,1\right\}$ for i=1,…,n such that $X_{i}={\left(X_{i0},X_{i1},\ldots ,X_{ip}\right)}^{T}$ and ${\left(X_{i1},\ldots ,X_{ip}\right)}^{T}∼N_{p}\left(0_{p},\Sigma \right)$, $\Sigma ={\left[\rho^{\left|i-j\right|}\right]}_{i,j=1,\ldots ,p}$ and ρ∈(−1,1). Response function is $q\left(x\right)=q_{L}\left(x^{3}\right)$ for x∈R, s={1,2} and $\beta_{s}={\left(1,1\right)}^{T}$. This means that:

$$P\left(Y_{i}=1|X_{i}=x_{i}\right)=q\left(\beta_{s}^{T}x_{i,s}\right)=q\left(x_{i1}+x_{i2}\right)=q_{L}\left({\left(x_{i1}+x_{i2}\right)}^{3}\right)$$

This model in comparison to Model M1 does not contain monomials of $X_{i1}$ and $X_{i2}$ of degree higher than 1 in its set of predictors. We observe that this binary model is misspecified with respect to fitted family of logistic models, because $q\left(x_{i1}+x_{i2}\right)≢q_{L}\left(\beta^{T}x_{i}\right)$ for any $\beta \in R^{p+1}$. However, in this case, the linear regressions condition in Equation (32) is satisfied for *X*, as it follows normal distribution (see [5,7]). Hence, in view of Proposition 3.8 in [6], we have $s_{log}^{*}=\left\{1,2\right\}$ and $\beta_{log,s_{log}^{*}}^{*}=\eta {\left(1,1\right)}^{T}$ for some η>0. Parameters n,p,ρ as well as *L* were chosen as for Model M1.

### 6.3. Results for Models M1 and M2

**Figure 1:**
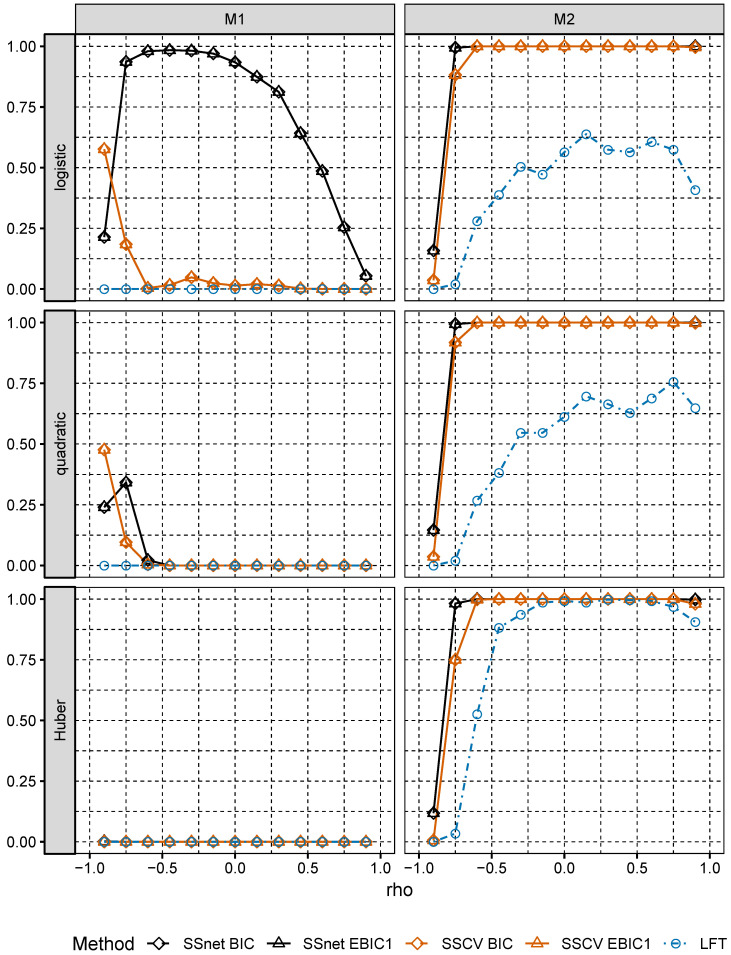
$P_{inc}$ for Models M1 and M2.

We first discuss the behavior of $P_{inc}$, $P_{equal}$ and $P_{supset}$ for the considered procedures. We observe that values of $P_{inc}$ for SSCV and SSnet are close to 1 for low correlations in Model M2 for every tested loss (see Figure 1). In Model M1, $P_{inc}$ attains the largest values for SSnet procedure and logistic loss for low correlations, which is because in most cases the corresponding family M is the largest among the families created by considered procedures. $P_{inc}$ is close to 0 in Model M1 for quadratic and Huber loss, which results in low values of the remaining indices. This may be due to strong dependences between predictors in Model M1; note that we have, e.g., $\mathrm{Cor}\left(X_{i1},X_{i8}\right)=3/\sqrt{15}\approx 0.77$. It is seen that in Model M1 inclusion probability $P_{inc}$ is much lower than in Model M2 (except for negative correlations). It it also seen that $P_{inc}$ for SSCV is larger than for LFT and LFT fails with respect to $P_{inc}$ in M1.

**Figure 2:**
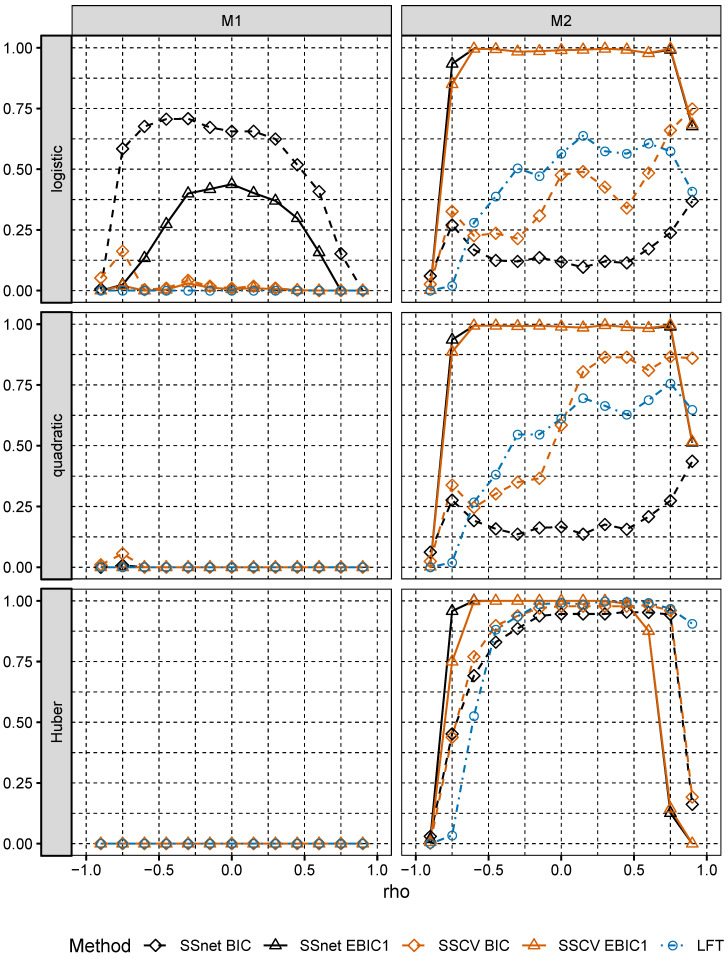
$P_{equal}$ for Models M1 and M2.

**Figure 3:**
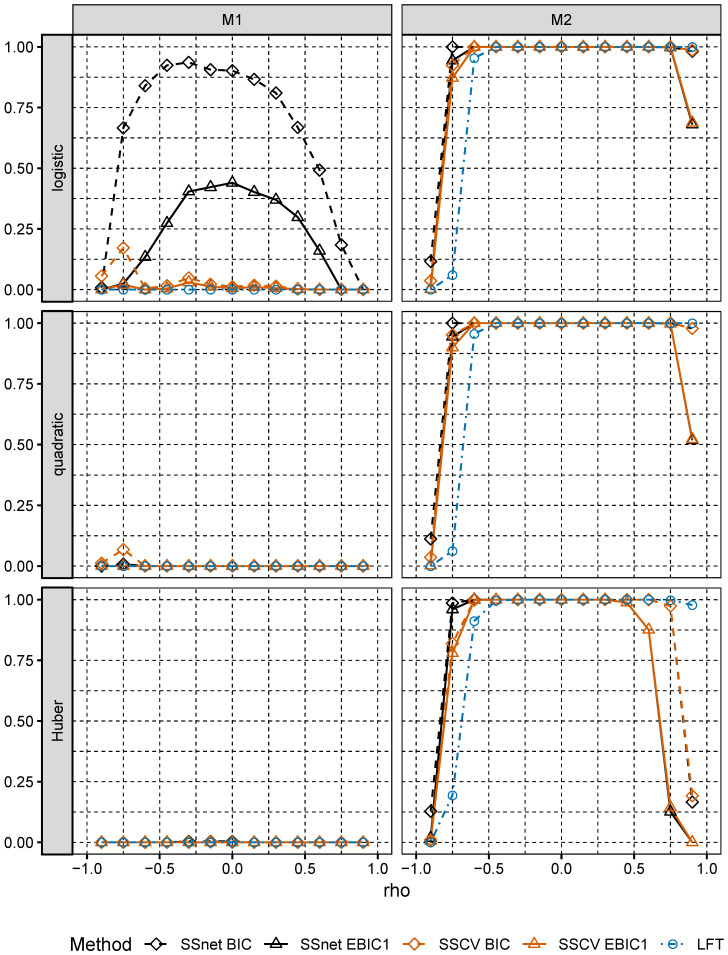
$P_{supset}$ for Models M1 and M2.

**Figure 4:**
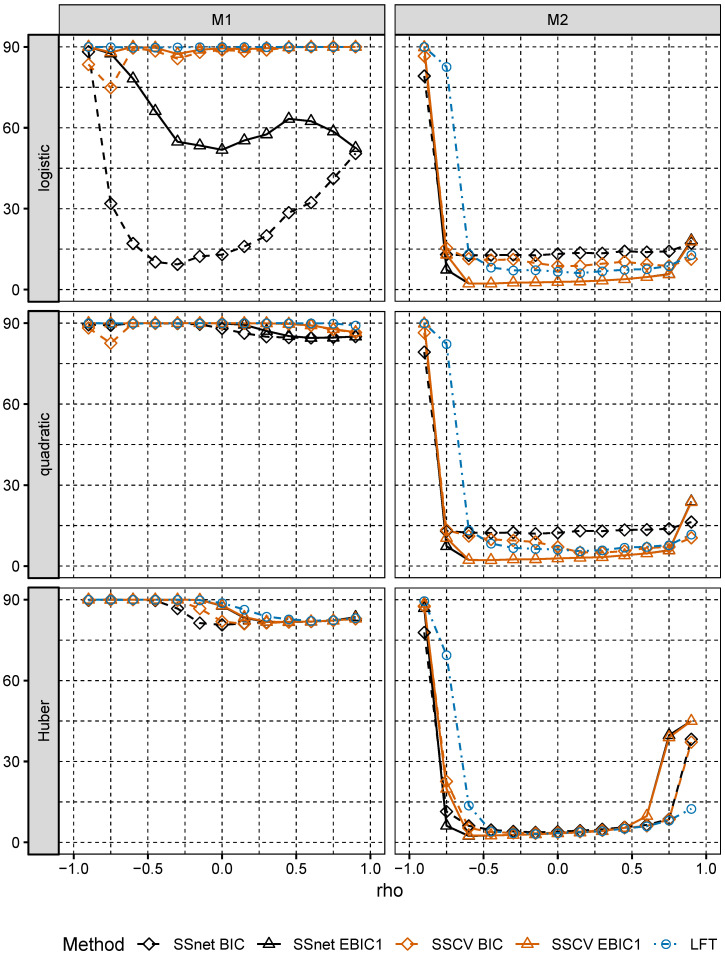
ANGLE for Models M1 and M2.

In Model M1, the largest values $P_{equal}$ are attained for SSnet with BIC penalty, the second best is SSCV with EBIC1 penalty (see Figure 2). In Model M2, $P_{equal}$ is close to 1 for SSnet and SSCV with EBIC1 penalty and is much larger than $P_{equal}$ for the corresponding versions using BIC penalty. We also note that choice of loss is relevant only for larger correlations. These results confirm theoretical result of Theorem 2.1 in [5], which show that collinearity holds for broad class of loss function. We observe also that, although in Model M2 remaining procedures do not select $s^{*}$ with high probability, they select its superset, what is indicated by values of $P_{supset}$ (see Figure 3). This analysis is confirmed by an analysis of ANGLE measure (see Figure 4), which attains values close to 0, when $P_{supset}$ is close to 1. Low values of ANGLE measure mean that estimated vector $\hat{\tilde{\beta }}\left({\hat{s}}_{k}^{*}\right)$ is approximately proportional to $\tilde{\beta }$, which is the case for Model M2, where normal predictors satisfy linear regressions condition. Note that the angles of $\hat{\tilde{\beta }}\left({\hat{s}}_{k}^{*}\right)$ and ${\tilde{\beta }}^{*}$ in Model M1 significantly differ even though Model M1 is well specified. In addition, for the best performing procedures in both models and *any* loss considered, $P_{equal}$ is much larger in Model M2 than in Model M1, even though the latter is correctly specified. This shows that choosing a simple misspecified model which retains crucial characteristics of the well specified large model instead of the latter might be beneficial.

In Model M1, procedures with BIC penalty perform better than those with EBIC1 penalty; however, the gain for $P_{equal}$ is much smaller than the gain when using EBIC1 in Model M2. LFT procedure performs poorly in Model M1 and reasonably well in Model M2. The overall winner in both models is SSnet. SSCV performs only slightly worse than SSnet in Model M2 but performs significantly worse in Model M1.

Analysis of computing times of the first and second stages of each procedure shows that SSnet procedure creates large families M and GIC minimization becomes computationally intensive. We also observe that the first stage for SSCV is more time consuming than for SSnet, what is caused by multiple fitting of Lasso in cross-validation. However, SSCV is much faster than SSnet in the second stage.

We conclude that in the considered experiments SSnet with EBIC1 penalty works the best in most cases; however, even for the winning procedure, strong dependence of predictors results in deterioration of its performance. It is also clear from our experiments that a choice of GIC penalty is crucial for its performance. Modification of SS procedure which would perform satisfactorily for large correlations is still an open problem.

## 7. Discussion

In the paper, we study the problem of selecting a set of active variables in binary regression model when the number of all predictors *p* is much larger then number of observations *n* and active predictors are sparse among all predictors, i.e., their number is significantly smaller than *p*. We consider a general binary model and fit based on minimization of empirical risk corresponding to a general loss function. This scenario encompasses the common case in practice when the underlying semi-parametric model is misspecified, i.e., the assumed response function is different from the true one. For random predictors, we show that in such a case the two-step procedure based on Lasso consistently estimates the support of pseudo-true vector $\beta^{*}$. Under linear regression conditions and semi-parametric model, this implies consistent recovery of a subset of active predictors. This partly explains why selection procedures perform satisfactorily even when the fitted model is wrong. We show that, by using the two-step procedure, we can successfully reduce the dimension of the model chosen by Lasso. Moreover, for the two-step procedure in the case of random predictors, we do not require restrictive conditions on experimental matrix needed for Lasso support consistency for deterministic predictors such as irrepresentable condition. Our experiments show satisfactory behavior of the proposed SSnet procedure with EBIC1 penalty.

Future research directions include considering the performance of SS procedure without subgaussianity assumption and for practical importance an automatic choice of a penalty for GIC criterion. Moreover, we note the existing challenge of finding a modification of SS procedure that would perform satisfactorily for large correlations is still an open problem. It would also be of interest to find conditions under which weaker than Equation (32) would lead to collinearity of β and $\beta^{*}$ (see [18] for different angle on this problem).

## Appendix A

Proof of Lemma 1:

**Proof.** Observe first that function $R_{n}$ is convex as ρ is convex. Moreover, from the definition of ${\hat{\beta }}_{L}$, we get the inequality:

(A1) $$W_{n}\left({\hat{\beta }}_{L}\right)=R_{n}\left({\hat{\beta }}_{L}\right)-R_{n}\left(\beta^{*}\right)\leq \lambda (||\beta^{*}{\left|\right|}_{1}-\left|\right|{\hat{\beta }}_{L}{\left|\right|}_{1}).$$

Note that $v-\beta^{*}\in B_{1}\left(r\right),$ as we have:

(A2) $$||v-\beta^{*}{\left|\right|}_{1}=\frac{\left|\right|{\hat{\beta }}_{L}-\beta^{*}{\left|\right|}_{1}}{r+\left|\right|{\hat{\beta }}_{L}-\beta^{*}{\left|\right|}_{1}}\cdot r\leq r.$$

By definition of $W_{n},$ convexity of $R_{n}$, Equation (A2) and definition of *S*, we have:

(A3) $$\begin{array}{cc} W(v) & =W\left(v\right)-W_{n}\left(v\right)+R_{n}\left(v\right)-R_{n}\left(\beta^{*}\right)\\ \; & \leq W\left(v\right)-W_{n}\left(v\right)+u\left(R_{n}\left({\hat{\beta }}_{L}\right)-R_{n}\left(\beta^{*}\right)\right)\leq S\left(r\right)+uW_{n}\left({\hat{\beta }}_{L}\right). \end{array}$$

From the convexity of $l_{1}$ norm, Equations (A1) and (A3), equality $\left|\right|\beta^{*}{\left|\right|}_{1}=\left|\right|\beta_{s^{*}}^{*}{\left|\right|}_{1}$, and triangle inequality, it follows that:

(A4) $$\begin{array}{cc} W\left(v\right)+{\lambda ||v||}_{1} & \leq W\left(v\right)+\lambda u||{\hat{\beta }}_{L}{\left|\right|}_{1}+\lambda \left(1-u\right)||\beta^{*}{\left|\right|}_{1}\\ \; & \leq S\left(r\right)+uW_{n}\left({\hat{\beta }}_{L}\right)+u\lambda (||{\hat{\beta }}_{L}{\left|\right|}_{1}-\left|\right|\beta^{*}{\left|\right|}_{1})+\lambda ||\beta^{*}{\left|\right|}_{1}\\ \; & \leq S\left(r\right)+\lambda ||\beta^{*}{\left|\right|}_{1}\leq S\left(r\right)+\lambda ||\beta^{*}-v_{s^{*}}{\left|\right|}_{1}+\lambda ||v_{s^{*}}{\left|\right|}_{1}. \end{array}$$

Hence,

$$\begin{array}{c} W\left(v\right)+\lambda ||v-\beta^{*}{\left|\right|}_{1}={\left(W\left(v\right)+\lambda ||v|\right|}_{1})+\lambda (||v-\beta^{*}{\left|\right|}_{1}-{\left||v|\right|}_{1})\\ \;\;\;\leq S\left(r\right)+\lambda ||\beta^{*}-v_{s^{*}}{\left|\right|}_{1}+\lambda ||v_{s^{*}}{\left|\right|}_{1}+\lambda (||v-\beta^{*}{\left|\right|}_{1}-{\left||v|\right|}_{1})=S\left(r\right)+2\lambda ||\beta^{*}-v_{s^{*}}{\left|\right|}_{1}. \end{array}$$

 □

We prove now Lemma A1 needed in the proof of Lemma 2 below.

**Lemma** **A1.**
*Assume that $S∼Subg(\sigma^{2})$ and T is a random variable such that |T|≤M, where M is some positive constant and S and T are independent. Then, $ST∼Subg(M^{2}\sigma^{2}).$*

**Proof.** Observe that:

$$Ee^{tST}=E\left(E\left(e^{tST}|T\right)\right)\leq Ee^{\frac{t^{2}T^{2}\sigma^{2}}{2}}\leq e^{\frac{t^{2}M^{2}\sigma^{2}}{2}}.$$

 □

Proof of Lemma 2.

**Proof.** From the Chebyshev inequality (first inequality below), symmetrization inequality (see Lemma 2.3.1 of [29]) and Talagrand–Ledoux inequality ([30], Theorem 4.12), we have for t>0 and ${\left(\varepsilon_{i}\right)}_{i=1,\ldots ,n}$ being Rademacher variables independent of ${\left(X_{i}\right)}_{i=1,\ldots ,n}$:

(A5) $$\begin{array}{cc} P(S(r)>t) & \leq \frac{ES(r)}{t}\\ \; & \leq \frac{2}{tn}E\underset{b\in R^{p_{n}}:b-\beta^{*}\in B_{1}\left(r\right)}{\sup}\left|\sum_{i=1}^{n}\varepsilon_{i}\left(\rho \left(X_{i}^{T}b,Y_{i}\right)-\rho \left(X_{i}^{T}\beta^{*},Y_{i}\right)\right)\right|\\ \; & \leq \frac{4L}{tn}E\underset{b\in R^{p_{n}}:b-\beta^{*}\in B_{1}\left(r\right)}{\sup}\left|\sum_{i=1}^{n}\varepsilon_{i}X_{i}^{T}\left(b-\beta^{*}\right)\right|. \end{array}$$

We observe that $\varepsilon_{i}X_{ij}∼Subg\left(\sigma_{jn}^{2}\right)$ in view of Lemma A1. Hence, using independence, we obtain $\sum_{i=1}^{n}\varepsilon_{i}X_{ij}∼Subg\left(n\sigma_{jn}^{2}\right)$ and thus $\sum_{i=1}^{n}\varepsilon_{i}X_{ij}∼Subg\left(ns_{n}^{2}\right).$ Applying Hölder inequality and the following inequality (see Lemma 2.2 of [37]):

(A6) $$E{\left|\left|\sum_{i=1}^{n}\varepsilon_{i}X_{ij}\right|\right|}_{\infty }\leq \sqrt{n}s_{n}\sqrt{2\ln(2p_{n})}\leq 2s_{n}\sqrt{n\ln(p_{n}\vee 2)}$$

we have:

$$\begin{array}{cc} \frac{4L}{tn}E\underset{b\in R^{p_{n}}:b-\beta^{*}\in B_{1}\left(r\right)}{\sup}\left|\sum_{i=1}^{n}\varepsilon_{i}X_{i}^{T}\left(b-\beta^{*}\right)\right| & \leq \frac{4Lr}{t}E\max_{j\in \{1,\ldots ,p_{n}\}}\left|\frac{1}{n}\sum_{i=1}^{n}\varepsilon_{i}X_{ij}\right|\\ \; & \leq \frac{8Lrs_{n}\sqrt{\log(p_{n}\vee 2)}}{t\sqrt{n}}. \end{array}$$

From this, Part 1 follows. In the proofs of Parts 2 and 3, the first inequalities are the same as in Equation (A5) with supremums taken on corresponding sets. Using Cauchy–Schwarz inequality, inequality ${\left||v|\right|}_{2}\leq \sqrt{\left|v\right|}{\left||v|\right|}_{\infty }$, inequality $\left|\right|v_{\pi }{\left|\right|}_{\infty }\leq {\left||v|\right|}_{\infty }$ for $\pi \subseteq \{1,\ldots ,p_{n}\}$, and Equation (A6) yields:

$$\begin{array}{cc} P(S_{1}\left(r\right)\geq t)\; & \leq \frac{4L}{nt}E\underset{b\in D_{1}:b-\beta^{*}\in B_{2}\left(r\right)}{\sup}\left|\sum_{i=1}^{n}\varepsilon_{i}X_{i}^{T}\left(b-\beta^{*}\right)\right|\\ \; & \leq \frac{4Lr}{nt}E\max_{\pi \subseteq \left\{1,\ldots ,p_{n}\right\},\left|\pi \right|\leq k_{n}}{\left|\left|\sum_{i=1}^{n}\varepsilon_{i}X_{i,\pi }\right|\right|}_{2}\\ \; & \leq \frac{4Lr}{nt}E\max_{\pi \subseteq \left\{1,\ldots ,p_{n}\right\},\left|\pi \right|\leq k_{n}}\sqrt{\left|\pi \right|}{\left|\left|\sum_{i=1}^{n}\varepsilon_{i}X_{i,\pi }\right|\right|}_{\infty }\\ \; & \leq \frac{4Lr\sqrt{k_{n}}}{nt}E{\left|\left|\sum_{i=1}^{n}\varepsilon_{i}X_{i}\right|\right|}_{\infty }\leq \frac{8Lr}{t\sqrt{n}}\sqrt{k_{n}}s_{n}\sqrt{\ln(p_{n}\vee 2)}. \end{array}$$

Similarly for $S_{2}\left(r\right)$, using Cauchy–Schwarz inequality, $\left|\right|v_{\pi }{\left|\right|}_{2}\leq \left|\right|v_{s^{*}}{\left|\right|}_{2}$, which is valid for $\pi \subseteq s^{*}$, definition of $l_{2}$ norm and inequality $E|Z|\leq \sqrt{EZ^{2}}\leq \sigma$ for $Z∼Subg(\sigma^{2})$, we obtain:

$$\begin{array}{cc} P(S_{2}\left(r\right)\geq t)\; & \leq \frac{4L}{nt}E\underset{b\in D_{2}:b-\beta^{*}\in B_{2}\left(r\right)}{\sup}\left|\sum_{i=1}^{n}\varepsilon_{i}X_{i}^{T}\left(b-\beta^{*}\right)\right|\\ \; & \leq \frac{4Lr}{nt}E\max_{\pi \subseteq s^{*}}{\left|\left|\sum_{i=1}^{n}\varepsilon_{i}X_{i,\pi }\right|\right|}_{2}\leq \frac{4Lr}{nt}E{\left|\left|\sum_{i=1}^{n}\varepsilon_{i}X_{i,s^{*}}\right|\right|}_{2}\\ \; & \leq \frac{4Lr}{nt}\sqrt{E{\left|\left|\sum_{i=1}^{n}\varepsilon_{i}X_{i,s^{*}}\right|\right|}_{2}^{2}}=\frac{4Lr}{nt}\sqrt{\sum_{j\in s^{*}}E{\left(\sum_{i=1}^{n}\varepsilon_{i}X_{ij}\right)}^{2}}\leq \frac{4Lr}{\sqrt{n}t}\sqrt{|s^{*}|}s_{n}. \end{array}$$

 □

Proof of Lemma 3.

**Proof.** Let *u* and *v* be defined as in Lemma 1. Observe that $||v-\beta^{*}{\left|\right|}_{1}\leq r/2$ is equivalent to $\left|\right|{\hat{\beta }}_{L}-\beta^{*}{\left|\right|}_{1}\leq r,$ as the function f(x)=rx/(x+r) is increasing, f(r)=r/2 and $f(||{\hat{\beta }}_{L}-\beta^{*}{\left|\right|}_{1})=||v-\beta^{*}{\left|\right|}_{1}$. Let C=1/(4+ε). We consider two cases: (i) $\left|\right|v_{s^{*}}-\beta_{s^{*}}^{*}{\left|\right|}_{1}\leq Cr$. In this case, from the basic inequality (Lemma 1), we have:

$$||v-\beta^{*}{\left|\right|}_{1}\leq \lambda^{-1}(W\left(v\right)+\lambda ||v-\beta^{*}{\left|\right|}_{1})\leq \lambda^{-1}S\left(r\right)+2||v_{s^{*}}-\beta_{s^{*}}^{*}{\left|\right|}_{1}\leq \bar{C}r+2Cr=\frac{r}{2}.$$

(ii) $\left|\right|v_{s^{*}}-\beta_{s^{*}}^{*}{\left|\right|}_{1}>Cr$. Note that $\left|\right|v_{s^{*c}}{\left|\right|}_{1}<\left(1-C\right)r,$ otherwise we would have $||v-\beta^{*}{\left|\right|}_{1}>r$, which contradicts Equation (A2) in proof of Lemma 1. Now, we observe that $v-\beta^{*}\in C_{\varepsilon },$ as we have from definition of *C* and assumption for this case:

$$\left|\right|v_{s^{*c}}{\left|\right|}_{1}<\left(1-C\right)r=\left(3+\varepsilon \right)Cr<\left(3+\varepsilon \right)||v_{s^{*}}-\beta_{s^{*}}^{*}{\left|\right|}_{1}.$$

By inequality between $l_{1}$ and $l_{2}$ norms, the definition of $\kappa_{H}\left(\varepsilon \right),$ inequality $ca^{2}/4+b^{2}/c\geq ab$, and margin Condition (MC) (which holds because $v-\beta^{*}\in B_{1}\left(r\right)\subseteq B_{1}\left(\delta \right)$ in view of Equation (A2)), we conclude that:

(A7) $$\begin{array}{cc} \left|\right|v_{s^{*}}-\beta_{s^{*}}^{*}{\left|\right|}_{1} & \leq \sqrt{|s^{*}|}\left|\right|v_{s^{*}}-\beta_{s^{*}}^{*}{\left|\right|}_{2}\leq \sqrt{|s^{*}|}||v-\beta^{*}{\left|\right|}_{2}\\ \; & \leq \sqrt{|s^{*}|}\sqrt{\frac{{\left(v-\beta^{*}\right)}^{T}H\left(v-\beta^{*}\right)}{\kappa_{H}\left(\varepsilon \right)}} \end{array}$$

(A8) $$\begin{array}{cc} \; & \leq \frac{\vartheta {\left(v-\beta^{*}\right)}^{T}H\left(v-\beta^{*}\right)}{4\lambda }+\frac{|s^{*}|\lambda }{\vartheta \kappa_{H}\left(\varepsilon \right)}\leq \frac{W(v)}{2\lambda }+\frac{|s^{*}|\lambda }{\vartheta \kappa_{H}\left(\varepsilon \right)}. \end{array}$$

Hence, from the basic inequality (Lemma 1) and the inequality above, it follows that:

$$W\left(v\right)+\lambda ||v-\beta^{*}{\left|\right|}_{1}\leq S\left(r\right)+2\lambda ||v_{s^{*}}-\beta_{s^{*}}^{*}{\left|\right|}_{1}\leq S\left(r\right)+W\left(v\right)+\frac{2|s^{*}|\lambda^{2}}{\vartheta \kappa_{H}\left(\varepsilon \right)}.$$

Subtracting W(v) from both sides of the above inequality and using the assumption on *S*, the bound on $|s^{*}|$, and the definition of $\tilde{C}$ yields:

$$||v-\beta^{*}{\left|\right|}_{1}\leq \frac{S(r)}{\lambda }+\frac{2|s^{*}|\lambda }{\vartheta \kappa_{H}\left(\varepsilon \right)}\leq \bar{C}r+\frac{2|s^{*}|\lambda }{\vartheta \kappa_{H}\left(\varepsilon \right)}\leq \left(\bar{C}+\tilde{C}\right)r=\frac{r}{2}.$$

 □

Proof of Remark 1.

**Proof.** Condition $\underset{n\to \infty }{lim inf}\frac{D_{n}a_{n}}{k_{n}\log\left(2p_{n}\right)}>1$ is equivalent to the condition that exists some u>0 that for almost all *n* we have:

$$D_{n}a_{n}-\left(1+u\right)k_{n}\log\left(2p_{n}\right)>0.$$

(1) We observe that, if

$$Aa_{n}-\left(1+u\right)k_{n}\log\left(2p_{n}\right)>0,$$

then the above condition is satisfied. For BIC, we have:

$$A\log n>\left(1+u\right)k_{n}\log\left(2p_{n}\right)>0,$$

which is equivalent to the condition (1) of the Remark. (2) We observe that using inequalities $k_{n}\leq C$, 2Aγ−(1+u)C≥0 and $p_{n}\geq 1$ yields for $n>2^{\frac{(1+u)C}{A}}$:

$$\begin{array}{c} A\left(\log n+2\gamma \log p_{n}\right)-\left(1+u\right)k_{n}\log\left(2p_{n}\right)\geq A\left(\log n+2\gamma \log p_{n}\right)-\left(1+u\right)C\log\left(2p_{n}\right)\\ =\left(2A\gamma -\left(1+u\right)C\right)\log p_{n}+A\log n-\left(1+u\right)C\log2\geq A\log n-\left(1+u\right)C\log2>0. \end{array}$$

(3) In this case, we check similarly as in (2) that

$$\begin{array}{c} A\left(\log n+2\gamma \log p_{n}\right)-\left(1+u\right)k_{n}\log\left(2p_{n}\right)\geq A\left(\log n+2\gamma \log p_{n}\right)-\left(1+u\right)C\log\left(2p_{n}\right)\\ =\left(2A\gamma -\left(1+u\right)C\right)\log p_{n}+A\log n-\left(1+u\right)C\log2>0 \end{array}$$

 □
